# Trust, Access, and the Division of Responsibility for Vaccine Confidence in Central and Eastern Europe: Paired Own-Country and Regional Ratings from a Multi-Stakeholder Professional Panel

**DOI:** 10.3390/vaccines14090819

**Published:** 2026-09-17

**Authors:** Teodor Cristian Blidaru, Alexandru Rafila, Diana Nastasă, David Sinclair, Petr Smejkal, Yasmin Maor, Ernest Kuchar, Mariano Votta, Luminița Vâlcea, Valeriu Gheorghiță

**Affiliations:** 1 Faculty of Medicine, “Carol Davila” University of Medicine and Pharmacy, Dionisie Lupu 37, 020021 Bucharest, Romaniavaleriu.gheorghita@umfcd.ro (V.G.); 2 National Institute for Health Services Management, Vaselor 31, 021254 Bucharest, Romania; 3 SMMR—Romanian Multidisciplinary Society of Resident Physicians, Mihail Kogălniceanu 39, 050103 Bucharest, Romania; 4 International Longevity Centre UK (ILC-UK), The Foundry, 17 Oval Way, London SE11 5RR, UK; 5 Department of Hygiene and Epidemiology, Institute for Clinical and Experimental Medicine (IKEM), Vídeňská 1958/9, 140 21 Prague, Czech Republic; 6 Infectious Disease Unit, E. Wolfson Medical Center, HaLochamim St. 62, Holon 5822012, Israel; 7 Gray Faculty of Medical and Health Sciences, Tel Aviv University, Ramat Aviv, Tel Aviv 6997801, Israel; 8 Department of Paediatrics with Clinical Assessment Unit, Medical University of Warsaw, Żwirki i Wigury 63A, 02-091 Warsaw, Poland; 9 Active Citizenship Network—Cittadinanzattiva, Via Imera 2, 00183 Rome, Italy; m.votta@cittadinanzattiva.it; 10 COPAC—Coalition of Organisations of Patients with Chronic Diseases in Romania, Str. Țepeș, Vodă 22, 021525 Bucharest, Romania; 11 “Prof. Dr. Agrippa Ionescu” Clinical Emergency Hospital, Str. Arh. Ion Mincu 7, 011356 Bucharest, Romania

**Keywords:** vaccination hesitancy, disinformation, attitude of health personnel, risk perception, health policy, healthcare disparities, public health, cross-sectional studies

## Abstract

Background/Objectives: Immunisation coverage gaps in Central and Eastern Europe (CEE) are commonly attributed to barriers of access. This study measured how the professionals who deliver immunisation services weigh competing barriers, whether their assessments differ when the referent is their own country rather than the CEE region as a whole, and how they rank candidate interventions on expected impact and near-term feasibility. Methods: Sixty-two professionals working in twelve countries and at the European level (47 based in CEE, 39 of them in Romania) completed a cross-sectional structured survey around a multi-stakeholder meeting in Bucharest (14 May 2026). Respondents rated 15 barriers twice, for their own country and for the CEE region; rated 16 interventions for the expected impact and 12–24-month feasibility; force-ranked their selections; and assigned the lead responsibility for five system functions. Results: Digital misinformation was the highest-rated barrier in every subgroup and at both geographic levels. The assessments differed by geographic referent: within respondents, trust and information barriers were rated more severe for their own country and access barriers more severe for the region (d_z = 0.52), a pattern attenuated but retained after standardising within each level; the same regional object was also described differently by respondents inside and outside CEE (d = 1.02). Across the 16 intervention-level means, the expected impact and feasibility were only weakly and imprecisely associated (r = 0.21, 95% CI −0.32 to 0.64), although, within respondents, the two were modestly correlated (r = 0.33). Continuing professional education for primary care ranked first on expected impact and led the forced-choice ranking in every subgroup. The lead responsibility was the clearest for professional training and least settled for equity monitoring. Conclusions: Barriers were assessed differently depending on whether the referent was the respondent’s own country or the region as a whole. These are professionals’ perceptions, not measured outcomes: access may rank low partly because the panel does not face it personally, and the findings do not place cost and structural determinants second. Regional strategies built on assumptions about neighbouring systems may misallocate attention, and the equity agenda this panel endorses rests on a monitoring function no actor owns.

## 1. Introduction

Vaccination represents one of the most cost-effective public health interventions currently available [1]; however, immunisation coverage gaps persist and, in several national contexts, appear to be widening [2,3]. The pattern is pronounced in Central and Eastern Europe (CEE), where paediatric coverage has declined in parts of the region since around 2010 [4,5,6,7], adult immunisation programmes remain limited in scope and infrastructure [5,8,9], and routine childhood immunisation coverage has remained below the European average in several countries [10]. Such disparities have conventionally been attributed to structural determinants of access—geographic distance, supply constraints, and cost [11]. In recent years, a substantial body of evidence points instead to vaccine confidence and trust in the health care system and physicians’ recommendations as more decisive determinants. Thus, vaccine uptake depends less on what people know than on whom they are prepared to believe [8,12].

Vaccine hesitancy is not a stable individual trait but a situational stance toward immunisation that varies with time, setting, and the specific vaccine [13]. It denotes an ambivalent position along a spectrum between acceptance and refusal, shaped by confidence in vaccine safety and providers, complacency about disease risk, and convenience of access [13]. Across CEE, this state is conditioned by a shared history: a post-communist inheritance of low institutional trust, uneven investment in adult and life-course immunisation, and health systems under strain, into which contemporary digital misinformation lands on already-weakened ground [14,15].

Two features of the policy problem follow. First, because the drivers of hesitancy are plural and interacting rather than singular, no single intervention is likely to suffice; population-level change is generally achieved through a portfolio of complementary measures acting at different levels of the system, each with its own realistic time horizon [16,17]. Second, choosing among candidate interventions requires that two considerations frequently collapsed into a single notion of “priority” be treated as analytically distinct: the magnitude of effect an intervention would produce once implemented, and the extent to which it can realistically be adopted and sustained within existing constraints [17,18,19]. Implementation science treats feasibility, acceptability, and appropriateness as outcomes conceptually distinct from effectiveness rather than as a discount applied to it, and validated instruments discriminate empirically between them [20,21]. Accordingly, each candidate intervention here was rated separately on expected impact and on feasibility, so that the basis of any ordering remains visible rather than buried in a composite score [19].

The groundwork was laid in 2024–2025, when a regional convening and the white paper that followed it inventoried what holds immunisation back across the region: false claims circulating online, public institutions that many citizens no longer credit, a thin understanding of what vaccines do, and services that are not equally reachable. Its closing argument was that the diagnostic phase had run its course [8,22]. A follow-up meeting in May 2026 turned to what should be done about it. The qualitative content of its discussion is synthesised separately, from the meeting transcript, generating a sister paper published as a perspective [23]; the present article reports the structured survey administered around that meeting. That paper, the 2025 white paper [22], and this study all arise from the same programme of work, and none of them is an independent source of support for the others. What a qualitative synthesis cannot provide is a comparable measurement of how professionals weigh these barriers and interventions against one another, and of whether their assessments differ when the referent is their own country rather than the region as a whole.

That last question motivates the study’s central design feature. Every barrier was rated twice by the same respondent, once for their own country and once for the CEE region as a whole. This makes it possible to ask not only which barriers professionals consider severe, but whether their assessment of the same barriers changes with the geographic referent. The question matters for a region whose policy response is increasingly coordinated: a regional strategy is built on shared assumptions about what neighbouring systems face, and those assumptions are rarely tested. The general-population literature on comparative and spatial optimism gives one reason to expect that such assessments may differ systematically by referent. People commonly judge conditions better “here” than “there”, though the bias is moderated by personal experience and perceived control and can attenuate or reverse for hazards that are locally salient [24,25,26]. Whether professional expertise confers immunity is not established; expert status is a weak predictor of judgement accuracy more generally [27]. Because the panel spans respondents based inside and outside CEE, the same region can additionally be compared as it is described from within and from without—a between-group contrast on a single shared object.

The study therefore addresses four questions. Which barriers do professionals judge most severe, and do those judgements differ systematically between their own country and the wider region, and between insiders and outsiders describing that region? Which interventions do they expect to have the greatest impact, and how does the expected impact relate to near-term feasibility? How does the equity dimension—the recognition that confidence operates differently for structurally disadvantaged groups—enter their assessments and their priorities? And who do they think should lead each of the main functions required to rebuild confidence, including the function of measuring whether equity gaps are closing? Throughout, the results are examined for robustness across the panel’s constituent groups rather than reported for the panel as an undifferentiated whole.

## 2. Materials and Methods

### 2.1. Design and Participants

A cross-sectional structured survey was conducted around the 14 May 2026 multi-stakeholder meeting “Building Vaccine Confidence in Central and Eastern Europe through Multi-Stakeholder Interventions”, held at the Romanian Parliament and organised by COPAC—Coalition of Organisations of Patients with Chronic Diseases in Romania—together with the Active Citizenship Network. The survey was distributed between 8 May and 30 June 2026, and 62 individuals responded. It was not disseminated publicly or advertised: the link was sent to participants in the 14 May meeting and to the direct professional networks of the organising partners, with a request that it be passed only to colleagues whose current work involves immunisation policy, delivery, advocacy, or research and who could therefore answer from direct professional knowledge. No a priori sample-size calculation was performed; the achieved sample is the number of eligible professionals who responded within the window, and the power it affords is discussed in Section 4.2. Respondents worked in twelve countries or at European level. Forty-seven were based in CEE (Romania 39, Poland 4, Slovenia 3, and Bulgaria 1) and fifteen outside it (Portugal 3, Cyprus 3, Malta 2, and single respondents from France, Greece, Israel, the Netherlands, and Spain, together with two respondents working at European level). The panel is therefore anchored in CEE, with a substantial minority of respondents who observe the region from outside it; the Romanian share is large, and the principal results are therefore also re-estimated, as sensitivity analyses, among CEE-based respondents, among respondents based outside CEE, and among Romanian respondents, with Romanian respondents excluded (Section 2.3). Eligibility was thus defined by professional role rather than by academic credential, and respondents self-identified their stakeholder category on entry. No threshold of seniority, publication record, or formal training in vaccine confidence was applied, and no respondent was screened out. The panel is therefore described throughout as a professional or stakeholder panel rather than as an expert panel in any credentialled sense, and it is non-probabilistic: there was no sampling frame, so no response rate can be calculated, and it is not a population-representative sample of any country or of the region. Composition is markedly uneven, which matters for how the aggregate ratings should be read. Civil-society and patient-organisation representatives (*n* = 25) and clinical health professionals (*n* = 21) were the largest groups, complemented by academics and researchers (6), public-health experts and epidemiologists (5), public-authority representatives (2), and single respondents in health communication, pharmacy, and independent vaccinology roles. The panel was experienced: 34 of 62 (54.8%) reported more than ten years in their field, and 54 of 62 (87.1%) reported at least occasional professional engagement with populations facing higher barriers to vaccination. Self-rated familiarity with formal vaccine confidence frameworks was, by contrast, modest and widely spread (median 2 of 5; 21 respondents selected the lowest point), which is addressed in the Limitations.

A qualitative synthesis of the discussion at the same meeting is reported separately [23]. The two studies share a framing event and an overlapping pool of participants, but use different instruments, data, and analytical questions. Section 4.2 explains why neither can be treated as independent corroboration of the other. Because the response window opened before and closed after the 14 May meeting, responses were received both before and after it: 37 were submitted on or before 14 May 2026 and 25 afterwards. Response date is a proxy for meeting exposure and not a record of it: attendance was not linked to responses, which were collected without identifiers, so respondents who answered after 14 May cannot be assumed to have attended and those who answered before cannot be assumed not to have been exposed to the meeting agenda. The principal within-respondent contrast holds in both windows, at +0.42 (95% CI +0.16 to +0.67; *p* = 0.002) among the earlier respondents and +0.47 (95% CI +0.08 to +0.86; *p* = 0.022) among the later ones, with no detectable difference between them (*p* = 0.83). Reporting follows the Consensus-Based Checklist for Reporting of Survey Studies (CROSS) [28]; the completed checklist is provided as Supplementary File S2. The instrument design draws on recommendations for structured expert elicitation and for consensus methods [29,30], although this was a single-round survey rather than an iterative consensus exercise.

### 2.2. Instrument

The full instrument, with every item, response option, and geographic instruction as administered, is provided as Supplementary File S1. It followed the analytical framework of the 2025 white paper and comprised six blocks: (A) respondent profile, including country, years of experience, level of operation, self-rated familiarity with vaccine confidence frameworks, and depth of professional engagement with populations facing higher barriers to vaccination; (B) validation of the 2025 white paper drivers and perceived direction of confidence since 2025; (C) 15 barriers, each rated on a 1–5 Likert scale (1 = minor, 5 = critical) at both the own-country and the CEE-region level, followed by an item on whether confidence challenges differ for groups facing structural barriers; (D) 16 candidate interventions, each rated for expected impact (1 = minimal, 5 = very high) and, separately, for feasibility within 12 to 24 months (1 = not feasible, 5 = fully feasible); (E) forced-choice rankings of the five most urgent barriers and of the five interventions judged most feasible and impactful for the respondent’s own country within 12 to 24 months, a flag for which of the respondent’s own selections were particularly important for reaching high-barrier populations, and assignment of a single preferred lead actor for each of five system functions; and (F) open-ended items. Barriers and interventions were each grouped into four thematic domains; for barriers, these were trust in institutions (C1), information and literacy (C2), access and system (C3), and attitudes, safety, and culture (C4). These domains follow the structure of the instrument, were fixed before any data were collected, and were not derived from the responses; no factor analysis was undertaken and the domain assignment should be regarded as conceptual rather than empirically validated. Forced-choice ranking was included alongside the rating scales because rankings compel discrimination between items in a way that rating scales, prone to non-differentiation and ceiling effects, do not [31]. All closed items in blocks C, D, and E4 were marked as required by the form, so those blocks are complete for all 62 respondents (the consent text stated that any question could be declined; in practice, a respondent who did not wish to answer a required item could only leave the form unsubmitted). The ranking grids in block E accepted up to five ranks: 55 respondents ranked five barriers and 54 ranked five interventions, the remainder ranked fewer, and all supplied ranks were scored. The form applied no technical control against multiple submissions; the closed-item response patterns were screened for duplicates and none was found. The instrument was administered in English through an online form (Google Forms) and was reviewed internally by the organising team before distribution; it was not piloted with external respondents, and no item wording was changed after distribution began.

### 2.3. Analysis

Ordinal ratings were summarised as means and the proportion of ratings at 4 or 5. Internal consistency of each rating block was assessed with Cronbach’s alpha, and additionally within each of the four thematic domains at each geographic level, since the domains, not the full item block, are the units of the principal contrasts; given the small number of items per domain, the mean inter-item correlation is reported alongside alpha. Means are reported to two decimal places, while differences and test statistics are computed from unrounded respondent-level data and may therefore differ by up to 0.01 from differences between the rounded means shown in the tables.

Because every respondent rated all 15 barriers at both geographic levels, and all 16 interventions on both dimensions, these contrasts are within-respondent and were analysed as paired comparisons. For each barrier, the own-country minus regional difference was computed per respondent and tested with the Wilcoxon signed-rank test, with the rank-biserial correlation as effect size; barriers were then aggregated into their four thematic domains and the domain-level contrast tested with a paired *t*-test and Cohen’s d_z. The first asymmetry hypothesis—that the own-country/region gap differs in sign between trust-and-information barriers and access barriers—was tested as a difference of differences within each respondent, which cancels any individual tendency to rate one geographic level more severely overall, using a paired *t*-test confirmed with the Wilcoxon signed-rank test. Because a difference in the spread of ratings between the two levels could produce that interaction without any difference in attribution, three further checks were run: the within-respondent standard deviation of the 15 ratings was compared between levels with a paired *t*-test; each item’s own-country-minus-region difference was related to the mean of its two ratings, which reduces the coupling that arises when one rating enters both terms; and the interaction was re-estimated after standardising each respondent’s ratings within each geographic level, which removes differences in both location and spread. The second asymmetry hypothesis concerns the regional ratings alone, which all respondents supplied for the same object: the trust-minus-access gap in each respondent’s regional ratings was compared between CEE-based and non-CEE-based respondents using Welch’s *t*-test with Cohen’s d, confirmed with the Mann–Whitney U test, and with the equivalent contrast on own-country ratings as a control.

Expected impact and feasibility were plotted against each other across the 16 interventions and the field split at the medians into quadrants, with the Pearson correlation between the item-level means and its 95% confidence interval reported. That correlation takes the intervention as the unit of analysis and therefore aggregates away the repeated-measures structure of the data, in which each respondent rated both dimensions for all 16 items. The association was therefore also estimated on the 992 paired ratings with a linear mixed model carrying crossed random intercepts for respondent and for intervention, fitted by restricted maximum likelihood with both factors entered as variance components, and with a repeated-measures correlation obtained by centring each respondent’s ratings on their own mean. Because the 16 interventions are a fixed list defined in advance rather than a sample drawn from a wider population, the item-level correlation is reported as a description of this set and not as an estimate for interventions in general. Forced-choice rankings were scored by assigning 5 points to a first-place rank down to 1 point for a fifth, summed across respondents, with the frequency of appearance in any respondent’s top five reported alongside. This linear weighting is conventional and descriptive rather than derived from any model of preference intensity, and a different weighting scheme could alter the precise ordering; both the weighted score and the top-five frequency are therefore reported so that the ranking can be read without relying on the weights. Agreement on lead responsibility was summarised as the modal share per function and, as a measure of dispersion, the Shannon entropy of the distribution across the five response options, H = −Σ p_i_ log p_i_, calculated with natural logarithms and divided by log 5 so that the result runs from 0 to 1 [32], where 0 denotes unanimity and 1 an even split. The association between depth of engagement with high-barrier populations and equity-related ratings was tested with Spearman’s rho across the ordered engagement categories; differences between stakeholder groups with Welch’s *t*-test and Cohen’s d; and associations between categorical responses with Fisher’s exact test. All principal results were re-estimated in the CEE-based and non-CEE-based subgroups and with Romanian respondents excluded. Open-ended responses were read and grouped into recurring themes by two authors and reconciled by discussion. Statistics were computed in Python 3.13 (pandas 3.0, SciPy 1.18; the linear mixed model in statsmodels 0.15, fitted by restricted maximum likelihood); all figures and tables were prepared in Microsoft Excel, except the quadrant plot of expected impact against feasibility, which was produced from the same summary data as described in Section 2.5.

Two-sided *p*-values are reported throughout. Three levels of inference are distinguished. The primary tests are the two prespecified asymmetry contrasts: the within-respondent difference-of-differences interaction and the between-group contrast on the regional ratings, each single planned comparison reported once, without adjustment. The re-estimation of these contrasts among CEE-based respondents, among respondents based outside CEE, among Romanian respondents, and with Romanian respondents excluded was not pre-specified: it was planned as a sensitivity analysis once the composition of the achieved panel was known, because Romanian respondents make up 63% of it. These four subsets overlap and are nested in the full sample (Romanian respondents are part of the CEE-based group, and the group with Romania excluded contains the non-CEE group), so they are not independent samples and no multiplicity adjustment is applied to them; they are reported to show whether the direction and approximate size of the primary estimates depend on the panel’s composition, and they are not treated as confirmation of the finding. The third level is exploratory: the fifteen per-item own-country/region comparisons are reported with uncorrected *p*-values for transparency and assessed as one family against a Benjamini–Hochberg false-discovery-rate correction, with the adjusted values given alongside the unadjusted ones in Table S2; the stakeholder and engagement comparisons are likewise reported without adjustment and read as descriptive. Per-item forced-choice rankings carry no inferential tests. Given the panel size, the study is powered only to detect moderate-to-large differences, and non-significant comparisons are reported as such and not as evidence of equivalence.

### 2.4. Ethics and Consent

Participation was voluntary and responses were collected without direct identifiers; no names, contact details, or institutional affiliations were recorded with the responses. One optional free-text item invited respondents to describe their role; because a small number of answers to it could, in principle, be indirectly identifiable in combination with country of practice, that field is reported only in aggregate and is withheld from any shared dataset. Before proceeding, every respondent was shown an information statement setting out the purpose of the survey, the absence of direct identifiers, and the intended use of the data in a peer-reviewed publication, and every respondent confirmed that statement before answering any question. All 62 responses are analysed together; no respondent group is treated differently.

Before publication, the Research Ethics Committee of the National Institute for Health Services Management (INMSS, Bucharest, Romania), with which one of the authors is affiliated, was asked to review the study, the full instrument with its consent text and an extended summary against the applicable legal framework (Law No. 206/2004 on good conduct in scientific research, Regulation (EU) 2016/679 and Law No. 190/2018). Its Determination of Exemption No. 3120 of 11 September 2026, issued at its meeting of 10 September 2026 in response to request No. 2977 of 2 September 2026, confirms that the study, as conducted, falls within the category of anonymous, voluntary, non-interventional surveys of professional opinion that are not subject to prior review by the Committee, and that the consent procedure and the handling of the free-text role field raise no ethical objection. The Committee noted that the assessment was requested after data collection had ended and that its determination classifies the study rather than constituting prior approval. The regimes that make such review mandatory are directed at other kinds of study. Regulation (EU) No 536/2014, which is directly applicable in Romania, governs clinical trials of medicinal products for human use and expressly does not extend to non-interventional studies. In Romania, the remit of the National Bioethics Committee for Medicinal Products and Medical Devices is medical research undertaken to advance therapy with medicinal products and medical devices, and Law No 95/2006 and Law No 46/2003 govern, respectively, medicinal products and the rights of patients receiving care. The present study is none of these: it enrolled no patients, administered no intervention, collected no health data, and asked professionals only for their assessments of policy priorities in their professional capacity. Respondents were informed of the purpose of the survey, of the intended publication, and of the fact that responses were collected without direct identifiers, and confirmed this before proceeding.

### 2.5. Use of Generative Artificial Intelligence

A generative AI assistant (Claude Opus 4, Anthropic, Model Opus 5) was used for language editing and phrasing, and to plot the quadrant figure of expected impact against feasibility from the mean values reported in the corresponding table. No image-generating model was used for that figure or for any other figure in this article, and no data, results, or references were generated by the assistant. The authors verified all analyses against the primary dataset, checked all cited sources against the published record, edited all AI-assisted output, and take full responsibility for the content of this article.

## 3. Results

### 3.1. Panel Endorsement of the 2025 Framework and Perceived Direction of Travel

Respondents largely endorsed the drivers carried over from the 2025 white paper: 38 judged them fully accurate and a further 18 mostly accurate but in need of some updates, so 56 of 62 (90%) affirmed the conceptual model, with 6 raising more fundamental reservations. The perceived direction of confidence since 2025 was mixed rather than uniformly negative: 27 respondents saw it as broadly unchanged, 13 reported some improvement, 14 reported some worsening, and 8 could not judge. Asked which developments had most influenced the confidence in the preceding twelve months, respondents most often named major misinformation campaigns and anti-vaccine narratives (33 of 62), followed closely by the introduction of new vaccines or vaccine programmes such as RSV, HPV catch-up and adult schedules (30), and political or institutional events affecting trust in authorities (27). Civil-society and patient-advocacy initiatives were named by 20 respondents, vaccine-preventable-disease outbreaks by 17, and improved or new public-health communication efforts by 13. The composition of that list is itself informative. The most frequently named influence was a threat to confidence, but the second was a programmatic expansion, and advocacy initiatives were named more often than outbreaks. The item asked only which developments had been most influential, not whether the influence was favourable or adverse, so these counts indicate salience and not direction: the introduction of new vaccines and schedules registered as strongly as the political events that erode trust, but the data do not show which way it moved the confidence. What they do show is that programmatic change is among the developments professionals notice most, alongside misinformation.

### 3.2. Information- and Trust-Related Barriers Rank Highest at Both Geographic Levels

**Figure 1:**
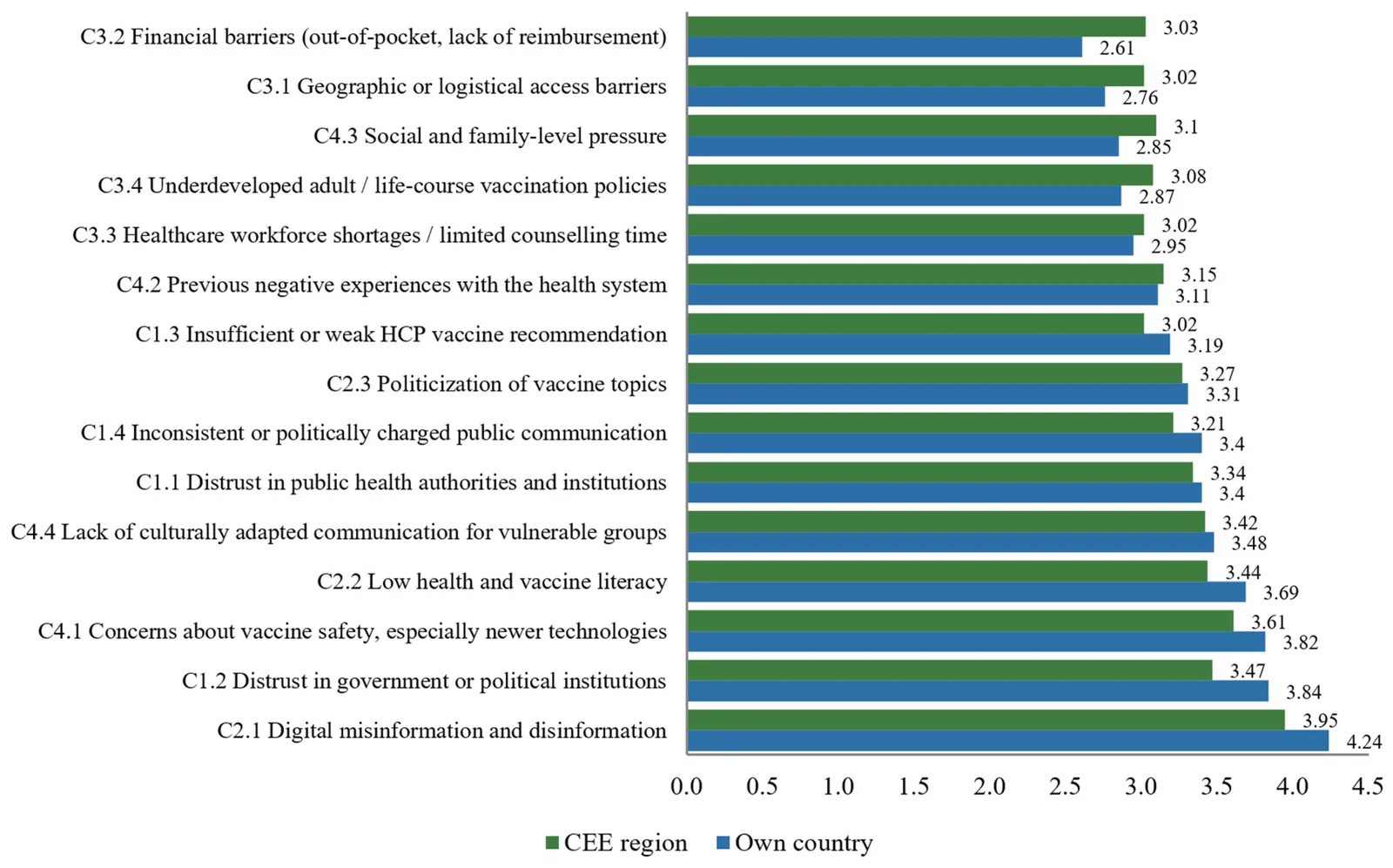
Mean barrier ratings at the own-country and CEE-region level (1 = minor, 5 = critical), ordered by country mean (N = 62).

The 15-item barrier scale was internally consistent at both levels of rating (Cronbach’s alpha 0.89 for own-country and 0.93 for regional ratings). Within domains, alphas were 0.70 for own-country and 0.84 for regional ratings in trust in institutions, 0.53 and 0.64 in information and literacy, 0.80 and 0.89 in access and system, and 0.74 and 0.81 in attitudes, safety, and culture; the corresponding mean inter-item correlations were 0.36 and 0.58, 0.29 and 0.38, 0.51 and 0.67, and 0.42 and 0.51. The information and literacy domain comprises only three items and is the least internally consistent of the four. At the country level, digital misinformation and disinformation was the highest-rated barrier (mean 4.24 of 5, SD 0.92; 81% rated it 4 or 5), followed by distrust in government or political institutions (3.84), concerns about the safety of newer vaccine technologies (3.82), and low health and vaccine literacy (3.69). The lowest-rated barriers at the country level were financial barriers (2.61) and geographic or logistical access (2.76). Digital misinformation was the single highest-rated barrier in every subgroup examined—CEE-based and non-CEE-based respondents, Romanian respondents, and the panel with Romania excluded—and at both the national and the regional level, making it the one unambiguous point of agreement across the panel. The agreement that confidence challenges differ markedly between the general population and groups facing structural barriers was moderate-to-high (mean 3.87 of 5) and is examined in Section 3.6. Table S1 reports all items at both levels, and Figure 1 shows the two orderings side by side.

The regional ratings did not, however, simply track the national ones, and the two sets diverged in a systematic way that forms the subject of the next section.

### 3.3. Own-Country and Regional Ratings Diverge by Barrier Type: Two Convergent Asymmetries

**Figure 2:**
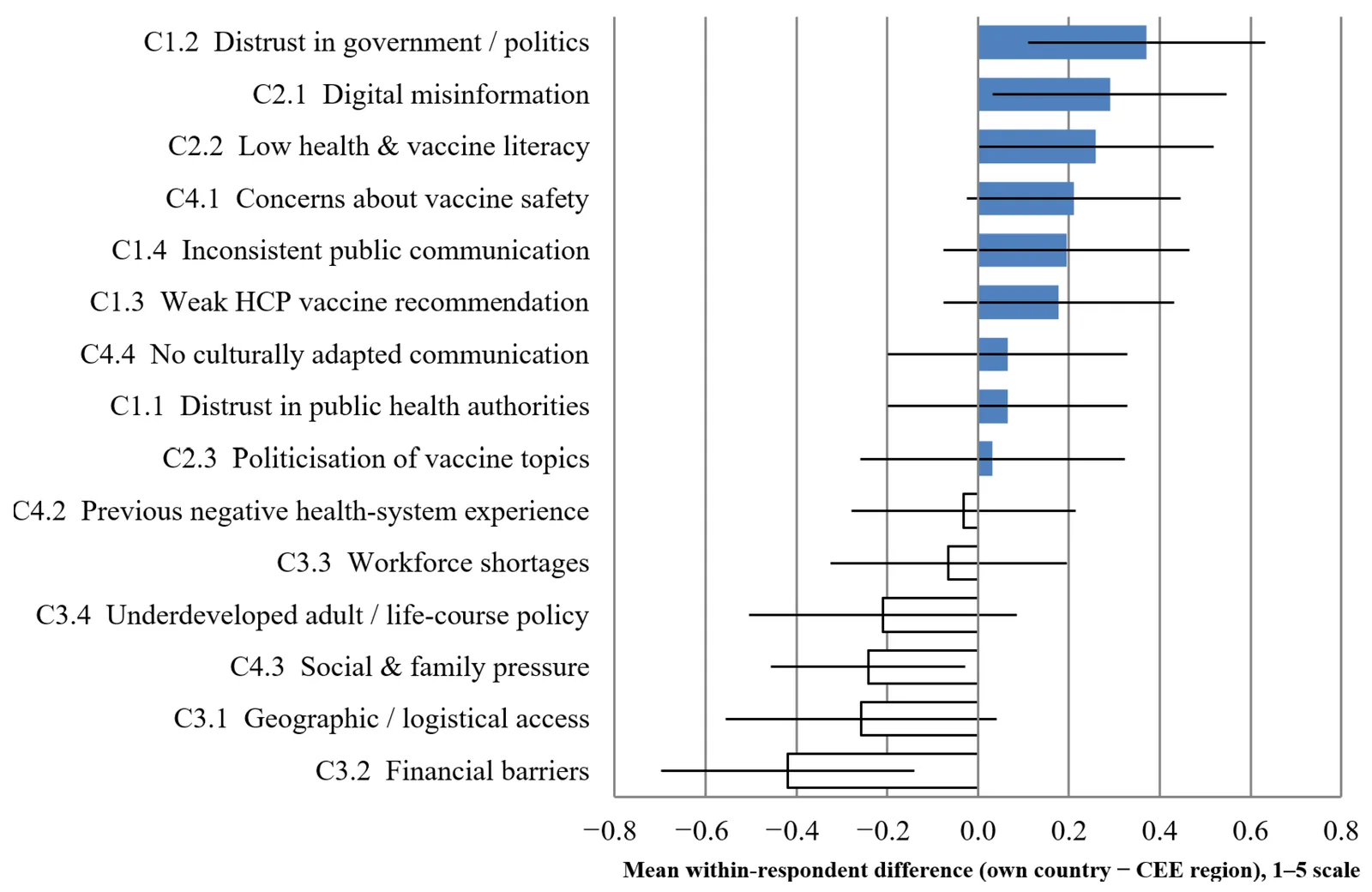
Within respondents: mean difference between own-country and CEE-region barrier severity ratings (own country minus region; 1–5 scale), ordered from the largest regional excess to the largest national excess. Positive values indicate a barrier rated more severe in the respondent’s own country. Error bars are 95% confidence intervals. Item codes match Table S1, where the full wording of each item is given (N = 62).

**Figure 3:**
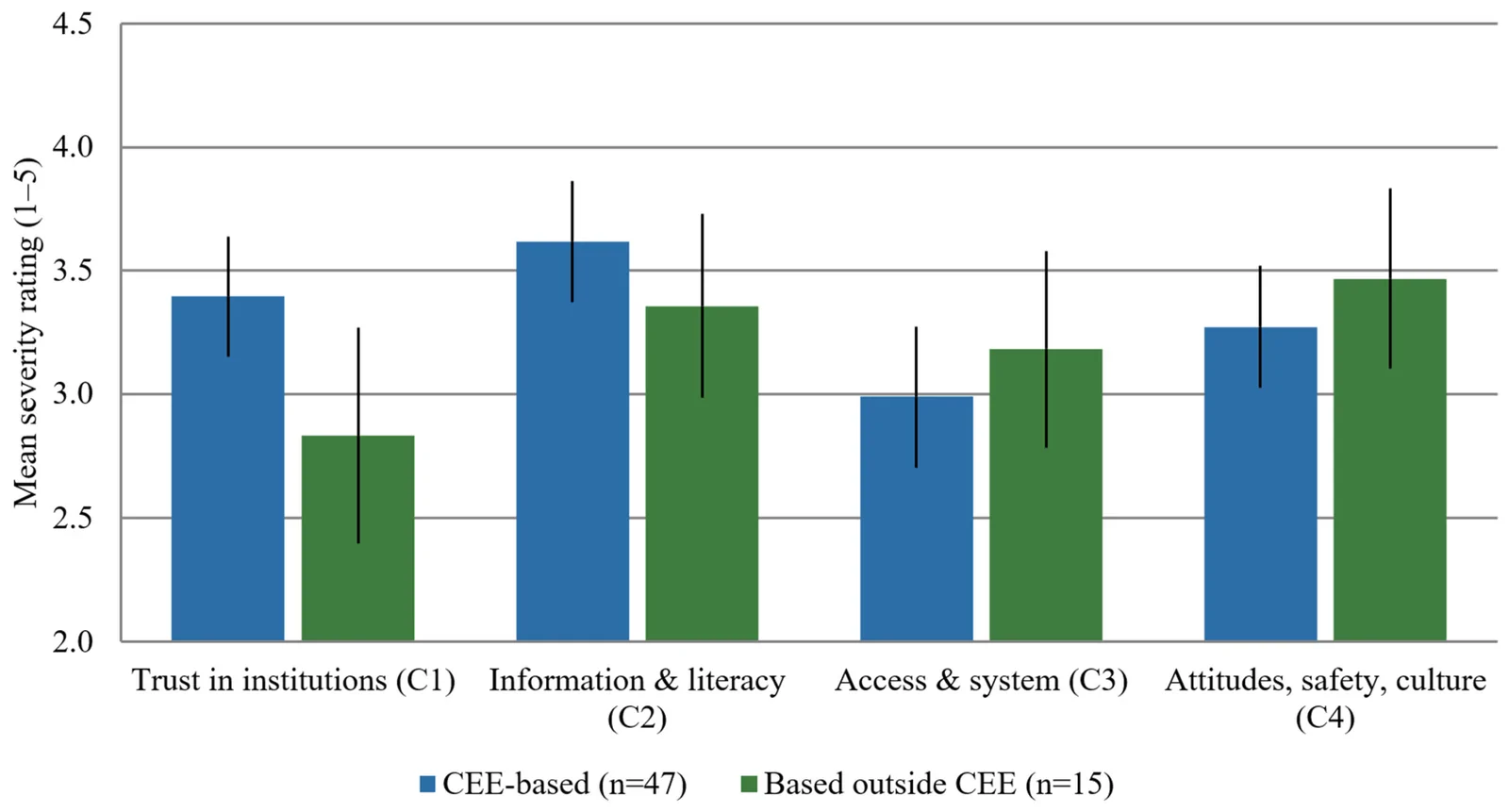
Between groups: mean severity ratings for the CEE region only—the same object for every respondent—by barrier domain, comparing respondents based in CEE (*n* = 47) with those based outside it (*n* = 15). Error bars are 95% confidence intervals (N = 62).

Every respondent rated all 15 barriers twice, once for their own country and once for the CEE region, so the two sets of ratings can be compared within each individual (Figure 2). Separately, because the panel includes respondents based inside and outside CEE, the regional ratings alone can be compared between groups who are describing the same object (Figure 3). The two comparisons ask different questions and are reported in turn; they share the regional ratings, so they are complementary rather than statistically independent tests.

The item-level picture is given first, to show where the effect sits; these fifteen comparisons are exploratory and none of them survives correction for multiple testing, as set out at the end of this section. Within respondents, trust- and information-related barriers were rated more severe at home than in the region. The largest single divergence was distrust in government or political institutions (own-country 3.84 versus regional 3.47; mean within-respondent difference +0.37; Wilcoxon *p* = 0.007; rank-biserial r = +0.56), followed by digital misinformation (4.24 versus 3.95; +0.29; *p* = 0.035) and low health and vaccine literacy (3.69 versus 3.44; +0.26; *p* = 0.054). Access- and system-related barriers ran in the opposite direction. Financial barriers showed the largest gap of that kind (2.61 versus 3.03; −0.42; *p* = 0.004; r = −0.56), followed by geographic or logistical access (2.76 versus 3.02; −0.26; *p* = 0.058) and underdeveloped adult and life-course vaccination policies (2.87 versus 3.08; −0.21). Aggregated to the domain level, trust in institutions (+0.20; paired t(61) = 2.53, *p* = 0.014, d_z = 0.32) and information and literacy (+0.19; t(61) = 2.36, *p* = 0.022, d_z = 0.30) were rated worse at home, while access and system barriers were rated worse in the region (−0.24; t(61) = −2.34, *p* = 0.023, d_z = −0.30).

A respondent might simply rate every item more severely at one level than the other. To remove that possibility, the asymmetry was tested as a difference of differences within each respondent. The interaction was clear: the trust-and-information gap exceeded the access gap by 0.44 scale points (95% CI +0.22 to +0.65; paired t(61) = 4.11, *p* = 0.00012, d_z = 0.52; Wilcoxon *p* = 0.0006), with 39 of 62 respondents showing the pattern, 17 the reverse and 6 tied. This interaction is the primary inferential test of asymmetry. In the sensitivity analyses described in Section 2.3, it kept its direction and a similar size: +0.38 among CEE-based respondents (t(46) = 3.42, *p* = 0.001), +0.62 among those based outside CEE (t(14) = 2.27, *p* = 0.040), +0.38 among Romanian respondents (*p* = 0.003), and +0.53 with Romanian respondents excluded (*p* = 0.015). These are overlapping subsets of the same panel, reported to show that the estimate does not depend on the Romanian majority, not independent replications of it. The fifteen per-item comparisons reported above are exploratory and are given to show where the effect is concentrated: none survives a Benjamini–Hochberg correction across the fifteen items (smallest q = 0.051, for financial barriers and for distrust in government; unadjusted and adjusted values for all fifteen are given in Table S2), and they should be read as descriptive rather than as independent confirmations.

One alternative account had to be excluded before this interaction can be read as a difference in attribution. Ratings of a less familiar, aggregate object may simply be less dispersed than ratings of a familiar one, pulling every high-rated item down and every low-rated item up without changing the grand mean. Because trust- and information-related barriers occupy the upper end of the severity ordering and access-related barriers the lower end, such compression would produce the observed interaction on its own. The data show that compression is real: the mean within-respondent standard deviation of the fifteen ratings was 0.99 at the own-country level and 0.67 at the regional level (paired t(61) = 7.20, *p* < 0.001, d_z = 0.91), while the two grand means were almost identical (3.30 and 3.27). Relating each item’s own-country-minus-region difference to the mean of its two ratings, rather than to either rating alone, reduces the coupling that arises when the same variable enters both sides of a comparison; on that formulation, the association is r = 0.85, against an expected value of about 0.5 under independence given the unequal spread of the two sets of item means, so, the higher an item’s overall severity, the larger its own-country excess, which is the compression itself and not independent evidence about attribution. The interaction was therefore re-estimated after standardising each respondent’s ratings within each geographic level, which removes the differences in both location and spread. It was attenuated but persisted, at +0.34 SD units (95% CI +0.10 to +0.57; paired t(49) = 2.92, *p* = 0.005, d_z = 0.41; Wilcoxon *p* = 0.020; twelve respondents with no variance at one or both levels cannot be standardised and drop out). Those twelve comprise nine CEE-based respondents, all in Romania, and three based outside the region (19% of the CEE-based group and 20% of the non-CEE group), so the exclusion is close to proportionate and does not favour either side of the between-group contrast. They span five stakeholder categories. In nine of the twelve, only the regional ratings carried no variance; the other three gave the same rating to every item at both levels and so contribute no own-country-minus-region difference to the unstandardised analysis either. No respondent showed the reverse pattern of flat own-country and varying regional ratings, which is itself consistent with reduced discrimination about the aggregate object. The twelve are also the respondents with the weakest raw asymmetry (mean interaction +0.15, against +0.50 among the fifty retained), so the standardised estimate describes the fifty respondents whose ratings varied at both levels; because standardisation is undefined for them, no re-estimate including them is possible, and the raw and standardised results should be read together. Part of the raw difference is thus attributable to reduced discrimination when rating the region, and part to a difference in the assessment by barrier type between the two referents; the second component does not disappear when the first is removed.

The fourth domain provides a useful contrast. Attitudes, safety, and culture showed a domain-level own-country/region difference of 0.00, but this is a cancellation rather than an absence of movement: concerns about newer vaccine technologies were rated worse at home (+0.21), while social and family pressure was rated worse in the region (−0.24), and the two offset each other. What the domain does not show is a coherent directional shift of the kind seen for trust, information, and access, which is what a uniform tendency to judge one’s own country more harshly would have produced across all four domains. Because this domain straddles the middle of the severity ordering, however, it cannot by itself separate attribution from dispersion; that separation rests on the standardised re-test reported above.

The second asymmetry concerns the regional ratings alone, which every respondent supplied for the same object. Respondents based in CEE and respondents based outside it described that region differently, and along the same axis. CEE-based respondents rated the region’s trust and information barriers above its access and system barriers, by 0.50 scale points (95% CI +0.31 to +0.69; *p* < 0.001). Among respondents based elsewhere, the corresponding gap was 0.13 points below zero, which is not distinguishable from no gap at all (95% CI −0.41 to +0.16; *p* = 0.36); the defensible reading is therefore that the ordering attenuates to null outside the region rather than that it reverses. The difference between the two groups was 0.63 scale points (95% CI +0.30 to +0.96; Welch t(29.1) = 3.86, *p* = 0.0006, Cohen’s d = 1.02; Mann–Whitney *p* = 0.0014). At the item level, CEE-based respondents rated regional distrust in public health authorities (3.51 versus 2.80, *p* = 0.019) and in government (3.64 versus 2.93, *p* = 0.029) markedly higher, while respondents based outside CEE rated the region’s underdeveloped adult and life-course vaccination policies higher (3.53 versus 2.94, *p* = 0.021).

Two features make this more than a restatement of the first asymmetry. The equivalent contrast on own-country ratings, where the two groups are describing different objects, was smaller and not statistically detectable at this sample size (+0.39; Welch t(21.5) = 1.35, *p* = 0.19); this is consistent with a divergence specific to judgements about the shared regional object, though, with 15 respondents outside CEE, it does not, on its own, establish one. The contrast survives removing Romania altogether: comparing the eight non-Romanian CEE respondents with the fifteen based outside CEE, the gap is +0.62 (*p* = 0.013), so it is not an artefact of the panel’s Romanian weighting. It is also the less exposed of the two comparisons to the dispersion account set out above, because both groups rated the same object, and it is, if anything, stronger after within-level standardisation (+0.85 SD units, *p* = 0.002). Both comparisons therefore show the same pattern: assessments differ by barrier type according to the geographic referent, with trust and information barriers weighted more heavily in the national assessment and access and system barriers more heavily in the aggregate regional one. The data show that the assessments diverge; they do not show where respondents locate each problem, and the candidate explanations are taken up in the Discussion.

### 3.4. Expected Impact and Near-Term Feasibility Diverge Across Interventions

**Figure 4:**
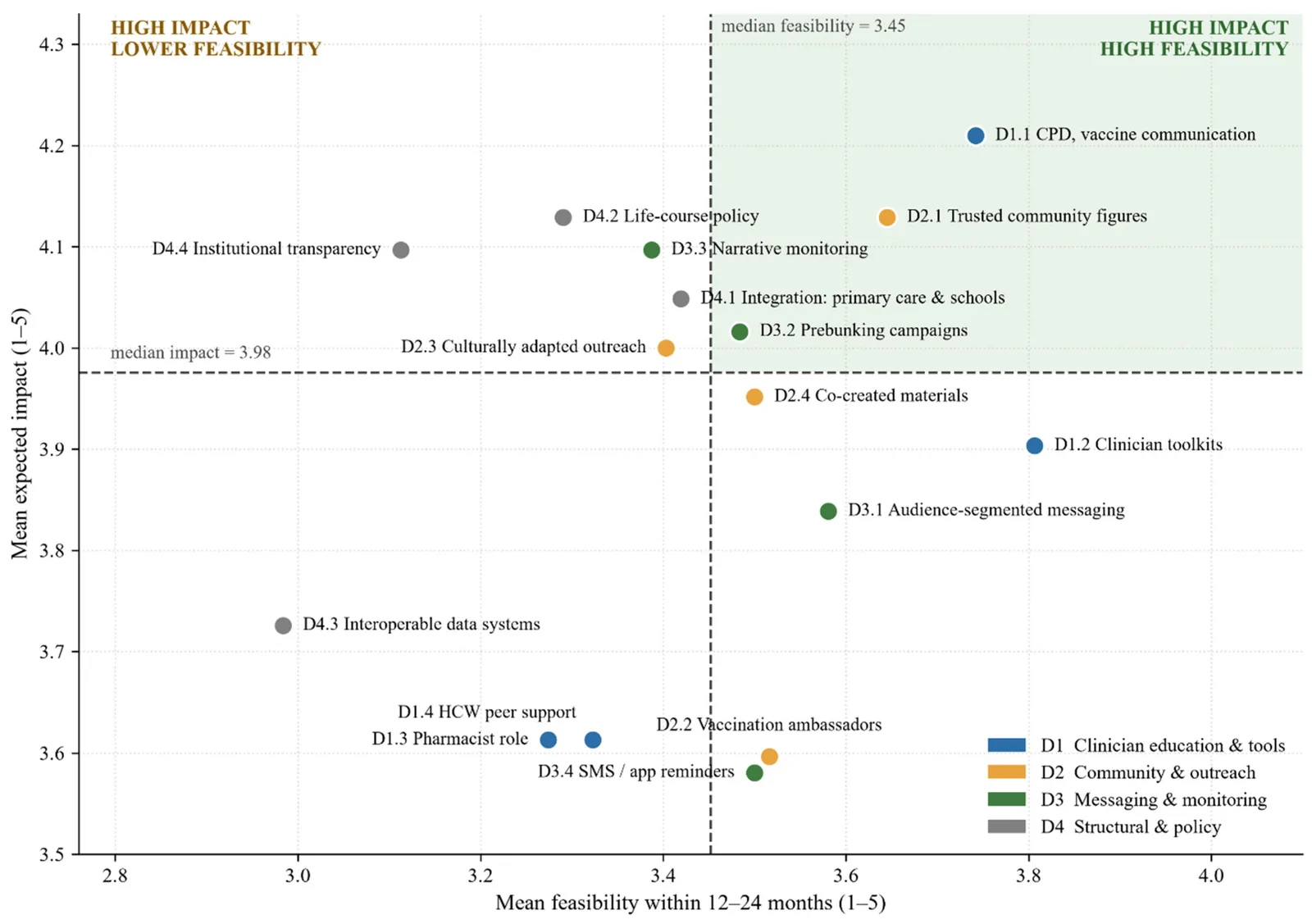
Expected impact plotted against feasibility within 12 to 24 months for the 16 candidate interventions. Dashed lines mark the medians of the two axes (expected impact 3.98, feasibility 3.45), which define the quadrants. Colours denote the four intervention domains (N = 62).

The 16 candidate interventions were rated separately for expected impact and for near-term feasibility. Across the 16 intervention-level means, the two were weakly and imprecisely associated (r = 0.21; 95% CI −0.32 to 0.64; *p* = 0.44; Spearman rho = 0.08); with only 16 points, the interval spans values from a moderate negative to a substantial positive association, so this analysis is compatible with no association but does not establish one. The intervention-level correlation also aggregates away the structure of the data. On the 992 paired ratings, a linear mixed model with crossed random intercepts for the respondent and intervention, with feasibility as the outcome and expected impact as the predictor, gave a clear positive association (slope 0.40, SE 0.03, 95% CI 0.33 to 0.46, *p* < 0.001; random-intercept variances 0.41 for respondent and 0.04 for intervention, residual 0.67), and the repeated-measures correlation was r = 0.33 (95% CI 0.27 to 0.39, *p* < 0.001); a similar value (r = 0.34) is obtained after centring on both the respondent and intervention means. Individual respondents therefore do tend to rate an intervention they consider impactful as somewhat more feasible, but the shared variance is about a tenth, so the two dimensions are related without being interchangeable. It is the ordering of the panel’s average ratings, not the within-respondent association, that the quadrant analysis below rests on. Both blocks were internally consistent (Cronbach’s alpha 0.93 for impact and 0.92 for feasibility). Splitting the field at the medians of both axes (expected impact 3.98, feasibility 3.45) produced four quadrants (Figure 4). Only three interventions combined an above-median impact with above-median feasibility: continuing professional education for primary care on vaccine communication (impact 4.21, feasibility 3.74), campaigns led by locally trusted community figures (4.13, 3.65), and proactive pre-bunking and counter-narrative campaigns (4.02, 3.48). Five were rated above the median on impact but below it on feasibility: the adoption of life-course immunisation policies (4.13, 3.29), real-time monitoring of vaccine narratives (4.10, 3.39), increased institutional transparency on vaccine decisions (4.10, 3.11—the largest impact-feasibility gap in the set), integration of vaccination into primary care and schools (4.05, 3.42), and outreach and culturally adapted programmes for vulnerable populations (4.00, 3.40). This quadrant is therefore not purely structural: it mixes system-level reform with the panel’s principal equity-facing measure. Notably, the intervention rated most feasible of all, communication toolkits and decision aids for clinicians (3.90, 3.81), fell just below the impact median, as did audience-segmented messaging (3.84, 3.58); investment in interoperable digital data systems fell below both medians (3.73, 2.98) and was rated the least deliverable of the 16. Table 1 reports every intervention and its gap; Tables S3 and S4 list the same interventions ordered by expected impact and by feasibility. Continuing professional education for primary care led the forced-choice ranking in every subgroup examined; on expected impact, it was first or second in three of the four subgroups and fifth among Romanian respondents (4.15, within 0.16 of that subgroup’s highest-rated intervention), and the adoption of life-course immunisation policies appeared in the top five of all four subgroup orderings. At the panel level, the expected impact and feasibility orderings differ (Spearman rho = 0.08 across the 16), whatever the within-respondent association, and a single composite priority score would conceal that difference.

### 3.5. Forced-Choice Priorities: Clinician Education and Trusted Community Messengers Lead

Required to name only their five most urgent barriers, respondents placed digital misinformation and distrust in public health authorities well ahead of the field, cited in the top five by 39 and 38 of 62 respondents, respectively (weighted scores 148 and 136), followed by low health literacy (31 mentions, 101 points) and distrust in political institutions (31 mentions, 92 points). On interventions, continuing professional education for primary care was again the clear front-runner (32 top-five mentions, 126 points), followed by campaigns led by locally trusted community figures and clinician-facing communication toolkits (26 mentions each, 84 and 80 points), proactive pre-bunking (17 mentions, 69 points), an expanded pharmacist role (23 mentions, 64 points), and culturally adapted outreach for vulnerable populations (22 mentions, 63 points). Asked which single intervention they would personally champion in the next six months, respondents most often named clinician education, community-based outreach through trusted local figures, and culturally adapted programmes for vulnerable groups. Three elicitation formats—a rating scale, a forced-choice ranking, and an open free-text question—therefore converge on the same two front-runners. The three formats were completed by the same respondents in one sitting and drew on the same item list, so this consistency is internal rather than independent: it shows that the ordering does not depend on which of the three question formats is read, not that it would survive a different instrument or a different panel. Convergence was closer for interventions than for barriers. The forced ranking placed distrust in public health authorities second, although that item stood joint sixth of fifteen on the rating scale (3.40), while concerns about the safety of newer vaccine technologies rated third (3.82) but did not enter the leading group under a forced choice. This is the discrimination the ranking format was included to obtain: asked to choose rather than to rate, respondents separated the barriers they treat as drivers from those they merely regard as serious.

### 3.6. Equity Is Recognised in Principle and Unevenly Translated into Priorities

Respondents broadly accepted that vaccine confidence operates differently for structurally disadvantaged groups. Asked whether confidence challenges in their country differ markedly between the general population and groups facing structural barriers—the item named rural populations, Roma communities, migrants, people on low incomes, and persons with disabilities—the mean rating was 3.87 of 5 (SD 1.03), with 39 of 62 respondents (63%) agreeing at 4 or 5 and none selecting the lowest point of the scale. Agreement was higher among CEE-based respondents (mean 4.00; 68% at 4 or 5) than among those based outside the region (3.47; 47%).

That recognition was reflected in how the two explicitly equity-facing interventions were rated. Outreach and culturally adapted programmes for vulnerable populations scored 4.00 on expected impact, eighth of the 16 interventions, and 3.40 on feasibility, placing it above the impact median and below the feasibility median; tailored materials co-created with vulnerable communities scored 3.95 and 3.50, falling just below the impact median and just above the feasibility median. Their impact–feasibility gaps (+0.60 and +0.45) were nonetheless not distinguishable from those of the other fourteen interventions when the gaps themselves were compared within respondents (mean difference +0.06; paired t(61) = 0.58, *p* = 0.56; Wilcoxon *p* = 0.83). Equity-facing measures were not judged to be categorically harder to deliver than the rest of the portfolio—only, like most of it, harder to deliver than they are valuable.

Proximity to the populations concerned changed what respondents saw. The perceived severity of the barrier “lack of culturally adapted communication for vulnerable groups” rose monotonically with the depth of professional engagement with high-barrier populations, from 2.00 among those reporting no such engagement, to 3.42 among those engaging occasionally, to 3.75 among those engaging regularly (Spearman rho = +0.27, *p* = 0.043). The same ordering appeared, without reaching significance, for the expected impact (3.50/3.95/4.25; rho = +0.22, *p* = 0.10) and the feasibility (2.75/3.37/3.69; rho = +0.22, *p* = 0.10) of culturally adapted outreach. The agreement that challenges differ for disadvantaged groups did not vary with engagement at all (rho = −0.004, *p* = 0.98). The abstract proposition is accepted regardless of proximity; the concrete judgement of how large the communication barrier is, and how tractable its remedy, is not. The subgroup reporting no engagement is small (*n* = 4) and this gradient should be treated as a hypothesis for larger studies rather than an established effect. The ordinal test uses the 58 respondents whose engagement could be ordered; the four who selected “not applicable to my role” are excluded because they sit outside that ordering, and they rated this barrier highest of any group (mean 4.50), so their exclusion does not favour the reported gradient.

Asked to flag which of their own top five interventions were particularly important for reaching high-barrier populations, 46 of 62 respondents flagged at least one, and the remaining 16 recorded that their selections applied broadly without specific relevance to vulnerable populations. Among the 39 respondents who agreed that challenges differ markedly for these groups, 31 (79%) flagged at least one equity-relevant intervention, compared with 15 of 23 (65%) among those who did not agree; this difference was not statistically significant (Fisher exact *p* = 0.24).

The free-text item asking for one realistic, locally relevant action to reach these populations within twelve months drew substantive answers from 47 of 62 respondents, and these converged on delivery models rather than on the message content: mobile community outreach teams combining nurses, community health workers, and mediators; the activation and, repeatedly, the payment of Roma and rural health mediators as vaccine liaisons; on-site vaccination at the point of contact rather than referral onward; systematic checking for missing doses during any clinical encounter; and the structured follow-up of families who do not return. Respondents in several countries named groups outside the prompt’s list, including women in prison and strictly observant religious communities. The recurring emphasis on paying mediators, rather than on designing new materials, locates the perceived constraint in financing and staffing rather than in communication craft.

### 3.7. Modal Agreement on Lead Responsibilities, Alongside Cross-Sectoral Overlap in Selected Functions

**Figure 5:**
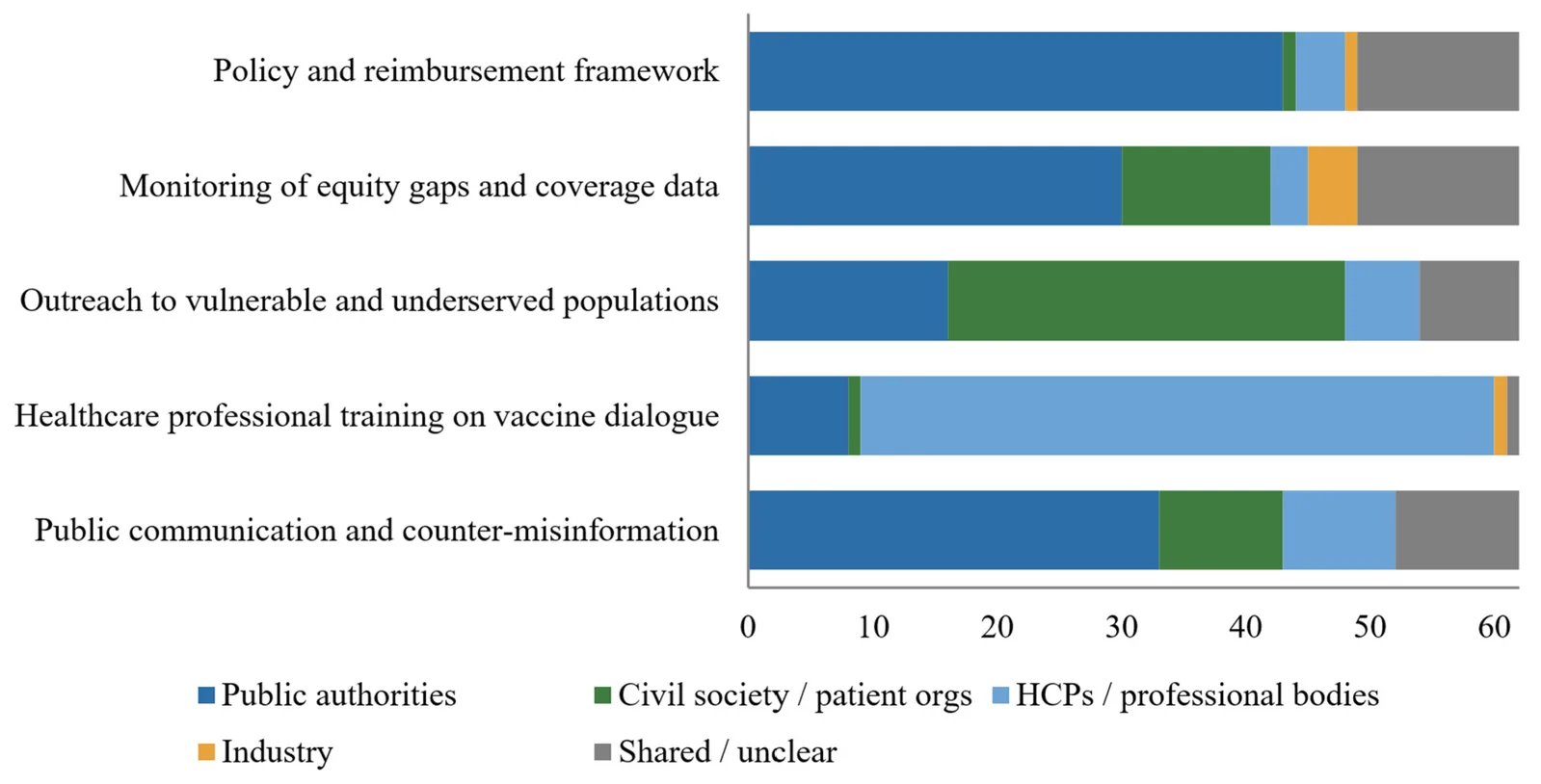
Preferred lead actor for each of five system functions (counts, N = 62). Normalised Shannon entropy of each distribution is given in parentheses, where 0 denotes unanimity and 1 an even split across the five response options.

Respondents were asked which single actor should take the lead on each of five system functions (Figure 5). Agreement was high for some functions and markedly lower for others. The training of health professionals on vaccine dialogue was the most consensual: 51 of 62 respondents (82%) assigned it to health professionals and their bodies, with only one respondent recording the function as shared or unclear (normalised entropy 0.39). The policy and reimbursement framework followed (43 of 62, 69%, to public authorities; entropy 0.55). Public communication and counter-misinformation was assigned to public authorities by 33 of 62 (53%; entropy 0.75), and outreach to vulnerable and underserved populations to civil-society and patient organisations by 32 of 62 (52%; entropy 0.73)—the only function for which civil society was the modal choice. Industry was almost never selected as the lead, receiving six nominations in total across all five functions. This allocation was notably stable across the panel: CEE-based and non-CEE-based respondents selected the same modal actor for all five functions, with modal shares differing by, at most, 15 percentage points, so the division of labour reported here is not specific to any one national context. The monitoring of equity gaps and coverage data was the least settled function of the five: public authorities were the modal choice but attracted under half the panel (30 of 62, 48%), 13 respondents recorded the function as shared or unclear, and the distribution across the five response options was the most dispersed measured (entropy 0.82); four of industry’s six nominations fell here. This matters because equity monitoring is the function on which the rest of the equity agenda depends. The outreach work that respondents confidently assigned to civil society cannot be targeted, or shown to have worked, without disaggregated coverage data that no actor was clearly assigned to produce. The panel converged on who should do equity work and diverged on who should measure it.

### 3.8. Themes from Open-Ended Responses

Open-text responses were read alongside the closed items and surfaced issues the scales did not capture. Five themes recurred across respondents and countries.

The first was provider-side hesitancy: several respondents stressed that reluctance is not only a public phenomenon but also sits within the health workforce, in the form of low conviction, fear of adverse events, and fear of legal responsibility, leading to unnecessary deferrals. Some HCP responses also pointed to a need to incorporate newer and recently approved adult vaccines into continuing medical education curricula. One respondent noted that provider uncertainty reinforces patient doubts and reduces uptake, and that confidence must therefore be built within the healthcare system and not only among the general public.

The second theme was organisational and access friction. Respondents described unclear vaccination pathways, booking difficulties, and fragmentation between primary care, pharmacies, and specialists. There are several proposed one-stop models, where assessment, prescription, and administration occur in a single visit, along with building vaccination into routine workflows and strengthening active recall systems. A number of responses also called for expanding prescribing and recommending rights beyond general practitioners, so that other medical specialties can vaccinate or refer patients for vaccination as part of routine care.

The third theme was transparency and liability: a cluster of responses called for a clear system of responsibility and no-fault compensation when a vaccine harms an individual, together with full public availability of vaccine data, including the risks and side effects, connecting the top-rated transparency intervention to a concrete governance demand.

The fourth theme concerned patient organisations and health literacy: respondents wanted these bodies formally embedded in national immunisation advisory processes, sustainable funding for patient-led education, and vaccine literacy strengthened through schools.

The fifth theme was local context and equity. Respondents pointed to several converging factors: fragmented vaccination calendars across the region, war-related strain on health systems and public trust, and religious, cultural, and needle-related fears. A further concern was the variability of vaccination coverage within a country, and, in particular, persistent under-vaccination among Roma and rural communities.

Finally, one respondent, based outside CEE, objected in free text to having been required to rate region-level items for a region to which they do not belong, and asked that those ratings be disregarded. No other respondent raised the point. This is a limitation of the instrument, discussed below; excluding that respondent leaves both asymmetries essentially unchanged (within-respondent interaction +0.42, *p* = 0.0002; between-group difference +0.64, *p* = 0.001).

### 3.9. Concordance Across Stakeholder Groups and Across the Panel’s Geography

Civil-society and patient-organisation representatives (*n* = 25) and clinical health professionals (*n* = 21) were compared across the four barrier domains and the four intervention domains (Table S5, Figure S1). No comparison reached statistical significance and all effect sizes were small. The largest was for access and system barriers, which civil-society respondents rated higher than clinical respondents (2.92 versus 2.55; Welch t(44.0) = 1.25, *p* = 0.22, d = 0.37), in the direction expected from their closer contact with under-served groups; trust-in-institutions barriers ran the other way (3.32 versus 3.54; Welch t(43.3) = −0.80, *p* = 0.43, d = −0.23). Across the intervention domains, differences ranged from 0.09 to 0.22 scale points, none significant, and both groups placed the structural and policy domain highest on expected impact. With these group sizes, the study can exclude only large differences: an effect of d = 0.37 would require roughly 116 respondents per group to detect reliably. The finding is therefore that no divergence large enough to reorder priorities was detected, not that the two constituencies hold identical views. The panel’s geography behaved similarly for everything except the regional ratings analysed in Section 3.3: CEE-based and non-CEE-based respondents agreed on the leading barrier, on the leading interventions and on the allocation of every system function, and diverged specifically and substantially in how they characterised the CEE region itself.

## 4. Discussion

This survey measured, on comparable scales, how a multi-country panel of professionals weighs the barriers and interventions that a regional discussion has already identified as relevant. Four results follow. The panel does not frame the problem primarily as one of access: misinformation, institutional distrust, and low literacy dominate both the rating scales and the forced prioritisation, while financial and geographic access rank lowest nationally, which is consistent with a confidence-centred reading of the region’s coverage gaps [8,12]. It converges on a small set of near-term interventions led by clinician communication training and locally trusted messengers. It endorses an equity agenda whose measurement function it does not clearly assign to anyone. And its assessment of the same barriers depends on the geographic referent, in two distinct ways: the same barriers were weighted differently for the respondent’s own country and for the region as a whole, and the same regional object was described differently from inside and outside it.

The referent-dependent result is the one that needs the most careful reading. Within respondents, trust- and information-related barriers were rated worse for the respondent’s own country and access- and system-related barriers worse for the wider region, consistently enough to survive a difference-of-differences test that removes any uniform tendency to judge one level more harshly (d_z = 0.52). That test does not, on its own, distinguish a difference in assessment from a difference in the spread of ratings, and the spread does differ: respondents discriminated markedly less when rating the region than when rating their own country. Standardising each respondent’s ratings within each level removes that difference, and the interaction survives it at a reduced size (+0.34 SD units, *p* = 0.005). The reading we adopt is therefore a compound one: part reduced discrimination about a less familiar object, and part a residual difference in how each kind of problem is weighed at the two levels, whose cause this design cannot identify. Between respondents, the same region was characterised differently by those inside and outside it (d = 1.02): insiders rated its trust deficits above its structural ones, while, among outsiders, that ordering flattened to no detectable difference. This second comparison is less exposed to the difference in spread, since both groups rated the same object, and it also survives standardisation (+0.85 SD units, *p* = 0.002; Section 3.3). The two contrasts are also not conceptually identical: for CEE-based respondents, the regional referent contains the national one, while, for the fifteen based outside the region, the two referents are separate places (Section 4.2). Several explanations are compatible with the pattern, and the design cannot choose between them. One is spatial optimism, documented in general populations across eighteen nations: respondents judged environmental conditions better locally than globally, and the optimism weakened where the local problem was objectively severe [26,33]. The moderator structure of comparative optimism would account for the direction, since biases driven by personal experience, salience, and perceived control act mainly on judgements about one’s own situation [24,25]; professionals experience their own system’s trust dynamics directly and infer other systems’ problems from the more visible categories of distance, cost, and infrastructure. A second, taken up below, is that respondents locate the responsibility for different kinds of problem at different levels. A third is coarser discrimination about the aggregate object, which the standardised re-test reduces but cannot exclude. The free-text objection reported in Section 3.8 is a further reason for caution about the regional ratings.

The practical consequence is for regional coordination, and it is not merely a caution about individual bias. Regional strategy documents, funding calls, and shared advocacy positions are built on assumptions about what neighbouring systems face, and the two asymmetries reported here indicate that such assumptions can be structured rather than random: if the pattern observed in this panel generalises, attention would tend to be directed towards structural remedies abroad and trust-building at home, whoever is speaking. For CEE specifically, this implies that regional initiatives designed by partners outside the region may systematically under-weight the institutional trust deficit that respondents inside it rate as paramount, while regional self-assessments may under-weight the adult and life-course policy gaps that outside observers rate more highly. Neither reading is validated here, and the asymmetry is a reason to compare notes instead of preferring one vantage point. A structural interpretation should also be considered alongside the perceptual one. The observed differences between barriers rated at home and at the regional level may not simply reflect differences in perception, but also the level at which respondents locate the responsibility for addressing them. Geographic and logistical constraints, as well as gaps in adult and life-course policy provision, are challenges that may require broader coordination, shared policies, and cross-border or European-level action. It is therefore plausible that respondents perceived these barriers as more prominent at the regional level. By contrast, deficits in trust are more directly shaped by individuals’ experience with their own healthcare system, national authorities, and the performance of domestic institutions, which may explain why they were rated as more severe at home. The observed pattern may therefore reflect, at least in part, a genuine division of institutional responsibility and not perceptual bias alone. However, because our study captures respondents’ perceptions rather than the objective institutional performance, the relative contribution of these two mechanisms cannot be determined without independent measures of performance at the national and regional levels. The corrective is not an exhortation but a structured exchange of what each system actually reports about itself— a form of peer learning among immunisation policymakers that is especially valuable in under-researched contexts, where contextual factors interact unpredictably [34]. A dual-level elicitation of the kind used here is inexpensive and could be embedded in any regional convening to make such divergences visible before they are built into strategy.

The prioritisation results point to an agenda that is unusually consistent across the panel. Continuing professional education for primary care on vaccine communication was first on expected impact, first in forced-choice ranking overall and within every geographic subgroup, and the most frequently named answer to an open question about what respondents would personally champion. Campaigns led by locally trusted community figures followed on each measure. Because all three formats were answered by the same respondents in one sitting, this is internal consistency rather than independent replication, but it does mean the ordering does not hinge on how the question was asked. The ordering is also compatible with what the behavioural literature already reports. Among the levers examined in a major synthesis of the psychological research on immunisation, few show as steady an association with uptake as a clear recommendation from the treating clinician [35], and where clinicians are themselves unconvinced or under-informed that recommendation weakens. A systematic review of provider vaccine perceptions traces that pattern through to a lower patient uptake [36]. Because respondents rated impact and feasibility separately, the interventions also sort into measures that are both valued and deliverable and measures rated equally valuable but considerably harder; the ordering of interventions on one axis was only a weak guide to their ordering on the other, even though individual respondents’ ratings on the two were modestly correlated. A strategy pursuing expected impact alone would front-load the hardest measures. This separation is not merely presentational: implementation research treats feasibility as an outcome distinct from effectiveness, and validated instruments discriminate between the constructs [20,21], while four-quadrant prioritisation grids of this general form are widely used in practice but rarely reported transparently [37]. A comparable divergence appears in a Delphi study of Australian immunisation stakeholders, who rated communication and accessible, affordable services as the two highest-priority challenges while identifying communication, alongside infrastructure and workforce constraints, as the most difficult aspects of their own work [38].

The equity results show a gap of a different kind, between principle and appraisal. Almost two-thirds of the panel agreed that confidence challenges differ markedly for structurally disadvantaged groups, and that the agreement was flat across respondents regardless of how much contact they had with such groups. What varied with contact was the concrete judgement: those working regularly with high-barrier populations reported a higher severity for inadequate culturally adapted communication, although the correlation was modest and the unengaged subgroup comprised only four respondents. The endorsement of the equity principle was near-universal in this panel; the appraisal of specific equity barriers was not. This is worth noting for any panel convened to set priorities, because a panel weighted away from frontline contact may endorse equity goals while under-rating the barriers that produce them. The free-text responses pointed consistently to delivery and financing over message design, and, above all, to paying community and Roma health mediators over new materials. The direction is well-chosen: undervaccination among Roma communities and migrant populations across Europe is well-documented and driven substantially by access and trust, not refusal [39,40,41], and lay and community health workers have demonstrated the effects on immunisation uptake [42,43]. The confidence attached to it, however, outruns the evidence. Mediator programmes in this region remain largely unevaluated for population-level impact [44], and evaluations of mobile vaccination outreach in England and the Netherlands both found the approach least effective in the most deprived and minority-ethnic neighbourhoods [45,46]. Two more recent studies bear on both points: vaccination coverage among Roma children remains below that of the surrounding population, with parental attitudes and access barriers both implicated [47], while a realist review of health-system interventions for minority populations in middle- and high-income countries finds that outcomes depend on whether services are redesigned around the population rather than merely offered to it [48]. Outreach is therefore not automatically equity-positive. That is not an argument against the panel’s preference, but it is an argument for commissioning such programmes with the evaluation built in instead of assuming the benefit.

The division-of-labour results give the agenda an implementable shape and expose where that shape breaks. Respondents assigned professional training to clinical bodies almost unanimously and policy and reimbursement to public authorities decisively; outreach to vulnerable populations was the one function civil society was given to lead. That this allocation was identical among CEE-based and non-CEE-based respondents suggests it reflects a broadly European professional consensus rather than a national administrative culture. Industry was almost never nominated as the lead, which is consistent with the position that industry participation in confidence work is tolerated in a contributing rather than a directing role, and that the credibility of the agenda depends on that boundary remaining visible; the same literature cautions that patient-advocacy organisations, to whom outreach is assigned here, face their own independence pressures [49,50]. The exception is instructive. The monitoring of equity gaps and coverage data attracted the lowest modal share, the most dispersed distribution of any function, and the largest share of industry nominations. An agenda that assigns delivery confidently and measurement ambiguously is structurally fragile, because disaggregated coverage data is what converts outreach from an activity into a demonstrable outcome. This is not only a problem of this panel: within-country inequality in vaccination coverage is inconsistently measured and reported in the published literature, and equity-oriented monitoring frameworks exist but are unevenly adopted [51,52]. Naming an owner for equity monitoring is the governance gap these data point to most directly.

Read together, the results translate into an agenda differentiated by the actor and by the confidence in the underlying evidence. For health professionals and their bodies, the near-term task is to scale continuing education on vaccine communication and to embed usable decision aids in routine consultations—the measure this panel rated highest on expected impact, among the most feasible, and assigned most clearly. For civil society and patient organisations, the priority is culturally adapted outreach to under-served groups, a function respondents assigned to them decisively, but one whose effect on the groups it targets is still thinly evidenced and should be measured, not presumed. For public authorities, the work is slower and foundational: transparency on how vaccine decisions are made, a movement towards life-course immunisation policy, interoperable data systems, an owner for equity monitoring, and a reimbursement framework that treats vaccination as a clinical service rather than an administrative act—adult immunisation financing remains markedly uneven across the European Union [53]. Respondents also attached two conditions to institutional transparency that the closed items did not anticipate: the full public availability of vaccine safety data, and a clear route to no-fault compensation where a vaccine causes harm. Both belong in the transparency agenda they endorsed, and neither is satisfied by publishing decision rationales alone. Because the quick wins and the structural measures were rated as comparably valuable and very differently feasible, and because they were assigned to different actors, these tracks can and should run in parallel, not in sequence.

### 4.1. Implications for Practice

Two measures were placed high on expected impact and on near-term feasibility at once, and were named again when respondents were asked what they would personally champion: continuing professional education for primary care on vaccine communication, and campaigns fronted by locally trusted community figures. Where a programme has to choose, those are the measures this panel would start with. A second group, made up of life-course immunisation policy, institutional transparency, and the integration of vaccination into primary care and schools, was rated equally valuable but markedly harder within two years, which is an argument for beginning them now rather than for deferring them until the quicker measures are complete. On equity, the free-text responses were more specific than the rating scales: respondents pointed to paying community and Roma health mediators, vaccinating at the point of contact rather than referring onward, checking for missing doses at any clinical encounter, and following up families who do not return. None of these is established as effective by this survey, and the evidence on outreach cautions against assuming they are; the practical recommendation is to commission them with evaluation attached. Finally, the one function the panel could not assign, monitoring of equity gaps and coverage data, is the function on which the rest of the equity agenda depends, and naming an owner for it is a decision that can be taken without new evidence.

### 4.2. Limitations

Several limitations qualify these results. The panel is non-probabilistic and self-selected, and Romanian, civil-society, and clinical respondents are over-represented; the ratings are the judgement of an engaged professional panel, not estimates from a representative population, and no country-level comparison is offered. The panel was also heterogeneous in professional background and stakeholder role, comprising clinicians, public-health specialists, civil-society and patient representatives, academics, and public-authority staff in markedly unequal numbers. Because these groups may differ systematically in their experiences, priorities, and perspectives, aggregate ratings may disproportionately reflect the views of the larger groups while obscuring those of less represented stakeholders. Meaningful stakeholder comparisons were feasible only for the two largest groups; therefore, the possibility that a professional background or stakeholder role influenced the observed ratings cannot be fully excluded. Because the Romanian share is large, the principal results were re-estimated as sensitivity analyses in the CEE-based and non-CEE-based subgroups and with Romanian respondents excluded, and each kept its direction; these are overlapping subsets of one panel rather than independent samples, and the non-CEE subgroup is small (*n* = 15, and *n* = 8 for non-Romanian CEE respondents), so the between-group contrast in Section 3.3, although large and confirmed by a non-parametric test, rests on limited numbers and requires replication. One respondent based outside CEE noted the difficulty of rating region-level items for a region in which they do not work. This is a genuine instrument limitation and it constrains how the regional ratings should be read. It cannot, however, explain the within-respondent asymmetry, which remained clear among CEE-based respondents rating their own region (+0.38, *p* = 0.001), a group to whom the objection does not apply, even though the effect was numerically larger among those based outside the region (+0.62). A further sampling limitation is that the survey was distributed to meeting participants and their professional networks without a fixed sampling frame, so the number of people invited is unknown and no response rate can be computed; the non-response bias cannot be assessed, and the panel should be read as a convenience sample of engaged professionals. These characteristics also limit the extent to which the findings can be generalized beyond CEE. Only fifteen respondents were based outside the region, and no individual non-CEE country contributed more than three participants. The findings should therefore be interpreted primarily as reflecting the perspectives of CEE-based professionals, complemented by those of a small and heterogeneous group of professionals based outside the region, rather than as representative of a broader European perspective.

This survey and the qualitative synthesis of the same 14 May 2026 meeting [23] draw on overlapping pools of participants and were prompted by the same regional agenda, and their conclusions converge on several points, notably the primacy of trust over access and the standing of clinician communication training and locally trusted messengers as near-term priorities. That convergence is reported here as a survey result in its own right, obtained through a structured instrument rather than through discussion, and it is mutually corroborating across two elicitation modes. It is not independent replication: because the respondent pools overlap and both exercises followed the same framing meeting, an agreement between them cannot be treated as confirmation by an unrelated source, and neither can be cited as external validation of the other. The two accounts also necessarily share vocabulary, since both refer to the interventions by the names under which the meeting and the survey instrument put them to participants. They do not agree at every point, and the disagreement is informative. The qualitative synthesis treats pharmacy-based vaccination and direct reimbursement as leading near-term levers, whereas this panel placed an expanded pharmacist role thirteenth of sixteen on expected impact and rated financial barriers the least severe of the fifteen. Two further features of the evidence base should be weighed here. The conceptual framework for the barrier and intervention lists is drawn from the 2025 white paper [22] and the ILC-UK report [8], neither of which was peer-reviewed; they are used as the source of the item structure that the panel was asked to rate, not as evidence for any claim made here. The substantive claim they encode, that the determinants of vaccine confidence are multi-domain and span trust in institutions, the information environment, access and service design, and attitudes and social influence, is itself well supported in the peer-reviewed literature, including an account of the behavioural, social, and contextual determinants of vaccine acceptance and demand written for the European region [54] and a synthesis of two decades of hesitancy research [55]; the grey-literature sources supplied the wording and the grouping of the items, not the evidence for the constructs. The white paper [22], the qualitative synthesis [23], and the present survey are three outputs of one project, published in the same year and drawing on overlapping groups of contributors; agreement among them is internal consistency within a single programme of work and cannot be read as independent corroboration from separate evidence streams. Discussion among a smaller group of speakers and a structured rating by a wider panel need not converge, and readers of both should treat the access-and-financing agenda as supported by the former rather than by the survey reported here.

The regional ratings were less dispersed than the own-country ratings across the fifteen items, consistent with reduced discrimination when judging a less familiar, aggregate object. Because trust- and information-related barriers occupy the upper end of the severity ordering and access-related barriers the lower end, such compression could, in principle, generate the within-respondent interaction without any difference in assessment by barrier type between the two referents. The between-group comparison is less exposed to this mechanism, since both groups rated the same object. The interaction survives standardisation within each geographic level (Section 3.3), but part of the raw effect reflects this reduced discrimination rather than a difference in attribution, so the standardised estimate (+0.34 SD units) rather than the raw difference should be taken as the size of the attributional component. The closest analogue among health professionals runs in one direction only: clinicians consistently judge antimicrobial resistance as a more serious problem nationally than in their own institution or practice [56,57]. That matches the direction observed here for access barriers but is the opposite of the direction for trust and information barriers, so the parallel shows that professionals’ assessments can depend on the referent without indicating which way they will run; it does not establish the mechanism, and we are not aware of an equivalent demonstration for vaccine confidence. A second and more fundamental limitation affects the within-respondent contrast itself: the comparison is not conceptually identical for all respondents. For the 47 CEE-based professionals, the own-country rating refers to a specific national setting and the regional rating to an aggregate that contains it, so the two are not independent referents; for the 15 respondents based outside the region, they are two separate places. Pooling both groups in the headline analysis therefore treats two different contrasts as estimates of one quantity. What the data establish is that assessments differ by barrier type between a national and an aggregate regional referent; they do not establish that respondents attribute structural problems to other countries, since a Romanian respondent comparing Romania with CEE as a whole is not comparing Romania with the rest of CEE. The between-group contrast, in which every respondent rates the same regional object, is the cleaner of the two on this point, although its non-CEE arm is small (*n* = 15) and its effect estimate correspondingly uncertain.

Further limitations concern measurement and power. The self-rated familiarity with formal vaccine confidence frameworks was modest across the panel, consistent with a group defined by practical, not academic, engagement with immunisation, but meaning the ratings should be read as an informed professional judgement rather than an appraisal against a common conceptual standard; expert status is, in any case, a weak predictor of judgement accuracy [27]. All measures are self-reported perceptions of severity, impact, and feasibility, not observed outcomes, and no external validation against coverage data was attempted; the asymmetry tests therefore establish that perceptions diverge, not which perception is closer to the truth. Adjudicating that would require pairing these judgements with measured national indicators, such as WHO/UNICEF coverage estimates, out-of-pocket vaccine costs, and the breadth of adult schedules; that pairing is the next step this design points to. The panel also rates barriers it does not itself face. Professionals who deliver immunisation services are not the population encountering cost, distance, time away from work, or competing demands, and a low severity rating for financial and geographic access records how those barriers appear from the delivery side, not how heavily they weigh on the people meeting them. A panel drawn from patients or from underserved communities could reasonably order the same fifteen items differently. Whether this panel over-weights trust and information barriers cannot be tested internally, because no independent measure of barrier severity exists for the same countries; the one internal signal is that the perceived severity of the culturally adapted communication barrier rose with a depth of engagement with high-barrier populations (Section 3.6), which is consistent with the proximity raising the weight given to barriers a respondent does not personally face. The stakeholder comparison used only the two largest categories and is powered to detect only large differences, so its null results are inconclusive, not confirmatory: with 25 and 21 respondents in the two largest groups, the comparison has 80% power only for differences of about d = 0.85, and the largest difference actually observed (d = 0.37) would require roughly 116 respondents per group to detect; the engagement gradient in Section 3.6 rests on a very small unengaged subgroup. The fixed lists of barriers and interventions, though grounded in the 2025 framework, constrain the response space, mitigated only partly by the open-ended items; the four thematic domains used for aggregation follow the structure of the instrument and were not empirically validated, so domain-level results should be read as summaries of their constituent items rather than as scores on established constructs. Taken together, these considerations support reading the results as a structured elicitation of professional judgement to guide prioritisation, not as a population-level estimate of vaccine-confidence conditions.

The instrument also limited what could be learned. Respondents were asked which actor should lead each system function but not why the responsibility for equity monitoring is so diffusely assigned, so the reasons behind the widest dispersion in the set, whether a contested mandate, absent capacity, or missing data infrastructure, remain unexamined. The barrier list also covers the supply side thinly: it captures weak clinician recommendation and workforce shortages but not clinicians’ own confidence in newer products, their perceived legal or professional exposure when recommending a vaccination, or the time and reimbursement available for a vaccination conversation. Continuing professional education ranks first in this panel’s priorities, and the instrument frames it around communication. That gap is visible in the study’s own data: provider-side hesitancy, fear of adverse events, and fear of legal responsibility appear in the first open-ended theme in Section 3.8, and calls for a no-fault compensation system to appear in the third, none of which had a closed item to attach to. Their absence from the ranked results is therefore an artefact of the item list rather than evidence of low salience, and an education recommendation confined to communication skills would address only part of what the panel itself identified. Ratings were also collected at a single time point, so nothing here speaks to the stability of these judgements, and priorities recorded at one moment may partly reflect what was salient at the time rather than a settled ordering. Three further constraints follow from the design. The instrument was reviewed internally before distribution but was not piloted with external respondents, so the item wording was not tested against how respondents outside the organising team read it, and whether the barrier items and the geographic instructions were read the same way across countries and professional backgrounds is unknown. The four domains are conceptual groupings fixed before data collection, not empirically derived constructs: the items in each were chosen because they name related policy problems, not because they were expected to load on a common latent variable. They are therefore closer to formative indices than to reflective scales, which is why no factor analysis was undertaken, and why the alpha values reported above should be read as descriptions of how closely the constituent items happened to move together rather than as validation of the grouping. Averaging within a domain is a summary of its items and is justified on that basis alone; it is most consequential for the three-item information and literacy domain, whose alpha of 0.53 at the own-country level indicates that its items behave fairly independently, so the domain mean should be treated as a convenient aggregate rather than as a score on a coherent construct. And, because the response window straddled the meeting, some responses were submitted before it and some afterwards, while attendance itself was not recorded; the principal contrast holds in both windows, with no difference detectable at this sample size, but the meeting may nonetheless have shaped later answers in ways this design cannot separate.

## 5. Conclusions

This panel of immunisation professionals, anchored in Central and Eastern Europe, ranks trust and information above access among the constraints on vaccine confidence, and converges on two near-term priorities: continuing professional education for primary-care professionals and campaigns led by locally trusted community figures. Assessments also depended on the geographic referent, with trust deficits rated more severe for the respondent’s own country and structural deficits more severe for the region as a whole; the same ordering appeared again when respondents inside and outside CEE rated the identical regional object. Part of the first pattern reflects the coarser discrimination about an aggregate, and the design cannot identify the mechanism behind the remainder, so it is reported as a difference in the assessment rather than as evidence of a particular cognitive bias. The practical implication does not depend on which explanation holds: what a regional strategy assumes about neighbouring systems cannot be presumed to match what those systems report about themselves, and the corrective is to compare the two directly before the assumptions are built into strategy. Three qualifications apply. They are professional perceptions, not measured outcomes. They come from people who deliver immunisation services rather than from those who live with the barriers being rated, so the low ranking of financial and geographic access records how those barriers look from the delivery side and does not place the cost and structural determinants second. And the panel, which endorses an equity agenda in principle and assigns the delivery of equity work confidently, leaves its measurement to no one in particular, which is the single clearest gap this survey identifies. The measurement approach used here, rating each barrier at two geographic levels and each intervention on two separate dimensions), and the expected impact and near-term feasibility, is inexpensive and can be reused wherever a group of stakeholders has to turn shared concern into an order of priorities.

## Figures and Tables

**Table 1:** Intervention ratings: mean expected impact, mean feasibility, and the impact–feasibility gap (positive = impact exceeds feasibility).

Intervention	Impact	Feasibility	Gap
D4.4 Increased institutional transparency on vaccine decisions	4.10	3.11	+0.98
D4.2 Adoption of life-course immunization policies	4.13	3.29	+0.84
D4.3 Investment in interoperable digital data systems	3.73	2.98	+0.74
D3.3 Real-time monitoring of vaccine narratives on social media	4.10	3.39	+0.71
D4.1 Integration of vaccination into primary care and schools	4.05	3.42	+0.63
D2.3 Outreach and culturally adapted programs for vulnerable populations	4.00	3.40	+0.60
D3.2 Proactive pre-bunking and counter-narrative campaigns	4.02	3.48	+0.53
D2.1 Local campaigns led by trusted community figures	4.13	3.65	+0.48
D1.1 Continuing education for primary care on vaccine communication	4.21	3.74	+0.47
D2.4 Tailored materials co-created with vulnerable communities	3.95	3.50	+0.45
D1.3 Expanded role for pharmacists in adult vaccination	3.61	3.27	+0.34
D1.4 Peer support/mentoring networks for healthcare workers	3.61	3.32	+0.29
D3.1 Audience-segmented messaging	3.84	3.58	+0.26
D1.2 Communication toolkits and decision aids for clinicians	3.90	3.81	+0.10
D2.2 Networks of vaccination ambassadors	3.60	3.52	+0.08
D3.4 SMS/app reminders and digital scheduling tools	3.58	3.50	+0.08

## Data Availability

The anonymised dataset supporting the findings is available from the corresponding author upon reasonable request. The free-text role-description field is withheld from any shared version, since a small number of responses to it could be indirectly identified when combined with country of practice; all analyses reported here can be reproduced without it.

## Supplementary Materials

The following supporting information can be downloaded at: https://www.mdpi.com/article/10.3390/vaccines14090819/s1, File S1: The complete survey instrument as administered; File S2: The completed CROSS checklist. Table S1: Barrier ratings by item at the own-country and regional level; Table S2, the fifteen item-level own-country versus region comparisons with unadjusted and Benjamini–Hochberg adjusted *p*-values; Tables S3 and S4, intervention ratings ordered by expected impact and by feasibility within 12 to 24 months; Table S5, mean domain ratings by stakeholder group; Figure S1, barrier severity by domain for civil-society and clinical respondents.
