# Supplementary material for: Trust, Access, and the Division of Responsibility for Vaccine Confidence in Central and Eastern Europe: Paired Own-Country and Regional Ratings from a Multi-Stakeholder Professional Panel
Source: Vaccines (Basel). 2026 Sep 17;14(9):819. doi: 10.3390/vaccines14090819 (PMC13611672; doi:10.3390/vaccines14090819)
Supplement: Supplementary file 1 [file vaccines-14-00819-s001.zip › suplement/Supplementary_File_S1_Survey_Instrument.pdf]

Supplementary File S1
Survey instrument as administered

"From Trust to Action — Building Vaccine Confidence in Central and Eastern Europe through Multi-Stakeholder Interventions." Self-administered online questionnaire (Google Forms), in English, open 8 May – 30 June 2026. Reproduced here in full: every item, response option, grid row and geographic instruction as presented to respondents. This document is a typeset transcription of the live form, not a screenshot of it.

1. Introduction and Informed Consent

Thank you for taking part in this consultation on vaccine confidence in Romania and Central/Eastern Europe.

PURPOSE: This survey is part of a structured consultation aimed at identifying priority barriers and feasible interventions to strengthen vaccine confidence in the region. Aggregated, anonymized results will inform a planned peer-reviewed publication. This work builds directly on the 2025 White Paper "Bridging the Confidence Gap: Strengthening Equitable Vaccination in Central and Eastern Europe."

WHAT YOU ARE ASKED TO DO: Complete this survey individually (~20 minutes). Respond from your own professional experience.

HOW YOUR RESPONSES WILL BE USED: Anonymized data will inform the resulting publication. Individual responses will not be linked to identifiable individuals in any publication.

VOLUNTARY PARTICIPATION: Your participation is entirely voluntary. You may decline any question or withdraw your responses at any time before publication. I have read the information above and understand the purpose of this survey I consent to my anonymized responses being used for the resulting peer-reviewed publication I confirm that I am responding in my professional capacity and not on behalf of any institution unless explicitly stated Vaccine Confidence in CEE through MultiStakeholder Interventions A multi-stakeholder consultation on vaccine confidence in Romania and Central/Eastern Europe. This survey takes approximately 20 minutes. * Indicates required question Informed consent — please tick all to continue *

1. Informed consent — please tick all to continue

Multiple selection.

- I have read the information above and understand the purpose of this survey
- I consent to my anonymized responses being used for the resulting peer-reviewed publication
- I confirm that I am responding in my professional capacity and not on behalf of any institution unless explicitly stated
- Vaccine Confidence in CEE through
- MultiStakeholder Interventions
- A multi-stakeholder consultation on vaccine confidence in Romania and Central/Eastern
- Europe. This survey takes approximately 20 minutes.
- * Indicates required question

Section A — Respondent Profile

type and country.

2. A1. Country of primary professional activity

Single choice from a drop-down list.

- Romania
- Bulgaria
- Poland
- Czech Republic

- Slovakia
- Israel
- Other (free text)

3. A2. Stakeholder type — please select the one that best describes your primary role

Single choice from a drop-down list.

- Healthcare professional — clinical (GP, pediatrician, specialist, nurse)
- Public health expert / epidemiologist
- Academic / researcher
- Civil society / patient organization representative
- Public authority / ministry / agency representative
- Pharmacist or pharmacy representative
- Health communicator / journalist
- Other (free text)

4. A3. Specific role or position

Free-text response.

5. A4. Years of professional experience in your current field

Single choice from a drop-down list.

- Less than 5 years
- 5–10 years
- 11–20 years
- More than 20 years

6. A5. Level at which your work is primarily situated (select all that apply)

Multiple selection.

- Local / community
- Regional (sub-national)
- National
- European / international

7. A6. Which of the following best describes your professional engagement with vaccination? (select all that apply)

Multiple selection.

- Direct clinical delivery of vaccines
- Patient or community education on vaccination
- Vaccine policy or strategy
- Research on vaccine confidence, hesitancy, or uptake
- Communication and media on vaccination
- Health workforce training
- Advocacy / civil society mobilization
- None of the above — vaccination is not my primary focus

8. A7. How familiar are you with established vaccine confidence frameworks (e.g., WHO SAGE, 3Cs/5Cs, BeSD, 5As)?

Linear scale from 1 to 5 (1 = not at all familiar, 5 = very familiar).

9. A8. In your professional work, do you regularly engage with populations facing higher barriers to vaccination (e.g., rural, Roma, migrant, low-income, persons with disabilities)?

Single choice from a drop-down list.

- Yes, regularly

- Yes, occasionally
- No
- Not applicable to my role

Section B — What Has Changed Since 2025?

The 2025 White Paper identified key drivers of low vaccine confidence in CEE:

- Inconsistent public health communication
- Digital misinformation and conspiracy narratives
- Limited health and vaccine literacy
- Limited trust in governments and health authorities
- Social and community pressure
- Concerns about vaccine safety, especially for newer technologies

We would like to know whether these still capture the situation in 2026.

10. B1. In your view, how well do the 2025 White Paper drivers above still capture the situation in your country in 2026?

Single choice from a drop-down list.

- They remain fully accurate
- They remain mostly accurate but need some updates
- They are partially out of date
- They miss something important

11. B2. If you indicated that the drivers need updates or miss something, please briefly explain what has changed or what is missing.

Free-text response.

12. B3. Since 2025, in your professional context, vaccine confidence has overall…

Single choice from a drop-down list.

- Improved meaningfully
- Improved slightly
- Stayed about the same
- Worsened slightly
- Worsened meaningfully
- I cannot judge

13. B4. In the past 12 months, which of the following developments have most influenced vaccine confidence in your country? (select up to 3)

Multiple selection.

- Measles or other vaccine-preventable disease outbreaks
- New vaccines or vaccine programs introduced (e.g., RSV, HPV catch-up, adult schedules)
- Political or institutional events affecting trust in authorities
- Major misinformation campaigns or anti-vaccine narratives
- Improved or new public health communication efforts
- Civil society or patient advocacy initiatives
- Changes in healthcare workforce capacity
- None of the above / no notable developments
- Other (free text)

Section C — Barriers to Vaccine Confidence

Below is a list of 15 potential barriers to vaccine confidence and uptake, grouped into four categories: C1 — Trust and communication; C2 — Information environment; C3 — Access and service design; C4 — Beliefs, experiences, and social influence.

We ask you to rate each as a barrier on a 1–5 scale, separately for: (a) your country, and (b) the broader CEE region as you perceive it.

The items are presented in two consolidated grids below.

Scale: 1 = Minor barrier • 3 = Moderate barrier • 5 = Critical barrier

C-A. Barriers in YOUR country

Rate each item below as a barrier in YOUR country.

14. Rate each item as a BARRIER in your country

Grid: 15 items, each rated on a 1–5 scale (1 = minor barrier, 5 = critical barrier).

- C1.1 Distrust in public health authorities and institutions
- C1.2 Distrust in government or political institutions
- C1.3 Insufficient or weak HCP vaccine recommendation
- C1.4 Inconsistent or politically charged public communication
- C2.1 Digital misinformation and disinformation
- C2.2 Low health and vaccine literacy
- C2.3 Politicization of vaccine topics
- C3.1 Geographic or logistical access barriers
- C3.2 Financial barriers (out-of-pocket, lack of reimbursement)
- C3.3 Healthcare workforce shortages / limited counselling time
- C3.4 Underdeveloped adult / life-course vaccination policies
- C4.1 Concerns about vaccine safety, especially newer technologies
- C4.2 Previous negative experiences with the health system
- C4.3 Social and family-level pressure
- C4.4 Lack of culturally adapted communication for vulnerable groups

15. Rate each item as a BARRIER across the CEE region

Grid: 15 items, each rated on a 1–5 scale (1 = minor barrier, 5 = critical barrier).

- C1.1 Distrust in public health authorities and institutions
- C1.2 Distrust in government or political institutions
- C1.3 Insufficient or weak HCP vaccine recommendation
- C1.4 Inconsistent or politically charged public communication
- C2.1 Digital misinformation and disinformation
- C2.2 Low health and vaccine literacy
- C2.3 Politicization of vaccine topics
- C3.1 Geographic or logistical access barriers
- C3.2 Financial barriers (out-of-pocket, lack of reimbursement)
- C3.3 Healthcare workforce shortages / limited counselling time
- C3.4 Underdeveloped adult / life-course vaccination policies
- C4.1 Concerns about vaccine safety, especially newer technologies
- C4.2 Previous negative experiences with the health system
- C4.3 Social and family-level pressure
- C4.4 Lack of culturally adapted communication for vulnerable groups

16. C5. To what extent do you agree: "In my country, vaccine confidence challenges differ markedly between the general population and groups facing structural barriers (e.g., rural, Roma, migrant, low-income, persons with disabilities)."

Linear scale from 1 to 5 (1 = strongly disagree, 5 = strongly agree).

17. C6. Are there any important barriers in your country that are NOT captured in the list above?

Free-text response.

Section D — Interventions: Impact × Feasibility

Below is a list of 16 potential interventions to strengthen vaccine confidence, grouped into four categories:

- D1 — Workforce and clinical interventions
- D2 — Community and civil society interventions
- D3 — Communication and digital interventions
- D4 — System and governance interventions

Each item is identified by its code (e.g., D1.1, D2.3) for easy reference.

Please rate each intervention on TWO dimensions: (a) expected IMPACT if implemented well, and (b) FEASIBILITY in your country within 12–24 months. To make this faster, the two ratings are split into two consolidated grids below.

18. Rate the EXPECTED IMPACT of each intervention if implemented well

Grid: 16 items, each rated on a 1–5 scale (1 = minimal, 5 = very high).

- D1.1 Continuing-education for primary care on vaccine communication
- D1.2 Communication toolkits and decision aids for clinicians
- D1.3 Expanded role for pharmacists in adult vaccination
- D1.4 Peer support / mentoring networks for healthcare workers
- D2.1 Local campaigns led by trusted community figures
- D2.2 Networks of vaccination ambassadors
- D2.3 Outreach and culturally adapted programs for vulnerable populations
- D2.4 Tailored materials co-created with vulnerable communities
- D3.1 Audience-segmented messaging
- D3.2 Proactive prebunking and counter-narrative campaigns
- D3.3 Real-time monitoring of vaccine narratives on social media
- D3.4 SMS/app reminders and digital scheduling tools
- D4.1 Integration of vaccination into primary care and schools
- D4.2 Adoption of life-course immunization policies
- D4.3 Investment in interoperable digital data systems
- D4.4 Increased institutional transparency on vaccine decisions

19. Rate the FEASIBILITY of each intervention in your country within 12–24 months

Grid: 16 items, each rated on a 1–5 scale (1 = not feasible, 5 = fully feasible).

- D1.1 Continuing-education for primary care on vaccine communication
- D1.2 Communication toolkits and decision aids for clinicians
- D1.3 Expanded role for pharmacists in adult vaccination
- D1.4 Peer support / mentoring networks for healthcare workers
- D2.1 Local campaigns led by trusted community figures
- D2.2 Networks of vaccination ambassadors
- D2.3 Outreach and culturally adapted programs for vulnerable populations

- D2.4 Tailored materials co-created with vulnerable communities
- D3.1 Audience-segmented messaging
- D3.2 Proactive prebunking and counter-narrative campaigns
- D3.3 Real-time monitoring of vaccine narratives on social media
- D3.4 SMS/app reminders and digital scheduling tools
- D4.1 Integration of vaccination into primary care and schools
- D4.2 Adoption of life-course immunization policies
- D4.3 Investment in interoperable digital data systems
- D4.4 Increased institutional transparency on vaccine decisions

20. D5. Are there any important interventions in your country or region that are NOT captured in the list above?

Free-text response.

Section E — Priority Rankings & Stakeholder Roles

This section consolidates your earlier ratings into priority rankings. In the ranking grids below, please tick exactly ONE cell per column — Forms will prevent you from selecting the same item for two different ranks. immunization policies

D4.3

Investment in interoperable digital data systems

D4.4 Increased institutional transparency on vaccine decisions

D4.3

Investment in interoperable digital data systems

D4.4 Increased institutional transparency on vaccine decisions

21. E1. From the list of barriers in Section C, please indicate your TOP 5 most urgent barriers in YOUR country, in order of priority.

Grid: 15 items listed; respondents could rank up to five of them (Rank 1 = highest), one item per rank.

- C1.1 Distrust in public health authorities and institutions
- C1.2 Distrust in government or political institutions more broadly
- C1.3 Insufficient or weak vaccine recommendation from healthcare professionals
- C1.4 Inconsistent or politically charged public health communication
- C2.1 Spread of digital misinformation and disinformation
- C2.2 Low public health literacy and low vaccine literacy
- C2.3 Politicization of vaccine topics in media and public discourse
- C3.1 Geographic or logistical access barriers (rural / underserved)
- C3.2 Financial barriers (out-of-pocket costs, lack of reimbursement)
- C3.3 Healthcare workforce shortages and limited counselling time
- C3.4 Underdeveloped adult vaccination policies / life-course frameworks
- C4.1 Concerns about vaccine safety, especially newer technologies
- C4.2 Previous negative experiences with the health system
- C4.3 Social and family-level pressure within close social groups
- C4.4 Lack of culturally adapted communication for vulnerable groups

22. E2. From the list of interventions in Section D, please indicate your TOP 5 most feasible AND impactful interventions for YOUR country in the next 12–24 months.

Grid: 16 items listed; respondents could rank up to five of them (Rank 1 = highest), one item per rank.

- D1.1 Continuing-education for primary care on vaccine communication
- D1.2 Communication toolkits and decision aids for clinicians
- D1.3 Expanded role for pharmacists in adult vaccination
- D1.4 Peer support and mentoring networks for healthcare workers
- D2.1 Local campaigns led by trusted community figures
- D2.2 Networks of vaccination ambassadors
- D2.3 Outreach and culturally adapted programs for vulnerable populations
- D2.4 Tailored materials co-created with vulnerable communities
- D3.1 Audience-segmented messaging
- D3.2 Proactive prebunking and counter-narrative campaigns
- D3.3 Real-time monitoring of vaccine narratives on social media
- D3.4 SMS/app reminders and digital scheduling tools
- D4.1 Integration of vaccination into primary care and schools
- D4.2 Adoption of life-course immunization policies
- D4.3 Investment in interoperable digital data systems
- D4.4 Increased institutional transparency on vaccine decisions and safety data

23. E2a. Of your top 5 interventions above (in E2), which (if any) do you consider PARTICULARLY important for reaching populations facing higher barriers to vaccination (rural, Roma, migrant, low-income, persons with disabilities)?

Multiple selection; the ranks refer to the respondent's own E2 ranking.

- Rank 1
- Rank 2
- Rank 3
- Rank 4
- Rank 5
- None specifically — these interventions apply broadly without specific relevance to vulnerable populations

24. E3. If you had to champion ONE intervention personally in the next 6 months, which one would it be — and why?

Free-text response.

25. E4. In multi-stakeholder work on vaccine confidence, which actor should TAKE THE LEAD on each of the following functions? (select one per row)

Grid: 5 functions, one actor selected per function from: Public authorities; HCPs / professional bodies; Civil society / patient orgs; Industry; Shared / unclear.

- Public communication and counter-misinformation
- Healthcare professional training on vaccine dialogue
- Outreach to vulnerable and underserved populations
- Monitoring of equity gaps and coverage data
- Policy and reimbursement framework

26. E5. What should civil society and patient organizations OWN in vaccine confidence work — and where do you see the limits of that role in a context where industry is also involved?

Free-text response.

Section F — Open Reflective Questions

captured by the structured items above. Please answer briefly — concise, candid responses are most useful.

27. F1. In your professional experience, what is the MOST UNDERESTIMATED cause of low vaccine confidence in your country — something that is rarely discussed in public debate but matters a lot?

Free-text response.

28. F2. What is ONE realistic short-term action (implementable in 6–12 months) that could meaningfully improve vaccine confidence in your country, and who should own it?

Free-text response.

29. F3. If we had this same conversation again in 12 months, what would you most want to be able to say has changed?

Free-text response.

30. F4. Thinking about populations who face the largest gaps in vaccine confidence and uptake in your country (e.g., rural, Roma, migrant, low-income, persons with disabilities, or other groups specific to your context): what is ONE realistic, locally-relevant action that could meaningfully reach them in the next 12 months?

Free-text response.
