# Supplementary material for: Trust, Access, and the Division of Responsibility for Vaccine Confidence in Central and Eastern Europe: Paired Own-Country and Regional Ratings from a Multi-Stakeholder Professional Panel
Source: Vaccines (Basel). 2026 Sep 17;14(9):819. doi: 10.3390/vaccines14090819 (PMC13611672; doi:10.3390/vaccines14090819)
Supplement: Supplementary file 1 [file vaccines-14-00819-s001.zip › suplement/Supplementary_File_S2_CROSS_Checklist.pdf]

Supplementary File S2 - Completed CROSS checklist

Consensus-Based Checklist for Reporting of Survey Studies (Sharma, A.; Minh Duc, N.T.; Luu Lam Thang, T.; et al. J. Gen. Intern. Med. 2021, 36, 3179–3187). Item wording is reproduced from the published checklist. Items that do not apply to a cross-sectional survey without a sampling frame are marked "Not applicable" with the reason given.

Note on numbering: the published Table 1 numbers the anonymity item 9c, while the fillable template numbers the same item 9b. The template numbering is the one editors normally see; both are shown below.

Section / topic	Item	Item description	Reported in
Title and abstract	1a	State the word "survey" along with a commonly used term in title or abstract to introduce the study's design.	Abstract (Methods: "cross-sectional structured survey"); the title names the design as paired ratings from a professional panel
Title and abstract	1b	Provide an informative summary in the abstract, covering background, objectives, methods, findings/results, interpretation/discussion, and conclusions.	Abstract
Background	2	Provide a background about the rationale of study, what has been previously done, and why this survey is needed.	Section 1
Purpose/aim	3	Identify specific purposes, aims, goals, or objectives of the study.	Abstract (Objectives); Section 1
Study design	4	Specify the study design in the "Methods" section with a commonly used term (e.g., cross-sectional or longitudinal).	Section 2.1
Data collection methods	5a	Describe the questionnaire (e.g., number of sections, number of questions, number and names of instruments used).	Section 2.2; File S1
Data collection methods	5b	Describe all questionnaire instruments that were used in the survey to measure particular concepts. Report target population, reported validity and reliability information, scoring/classification procedure, and reference links (if any).	Section 2.2
Data collection methods	5c	Provide information on pretesting of the questionnaire, if performed (in the article or in an online supplement). Report the method of pretesting, number of times questionnaire was pre-tested, number and demographics of participants used for pretesting, and the level of similarity of demographics between pre-testing participants and sample population.	Section 2.2; Section 4.2
Data collection methods	5d	Questionnaire, if possible, should be fully provided (in the article, or as appendices or as an online supplement).	File S1
Sample characteristics	6a	Describe the study population (i.e., background, locations, eligibility criteria for participant inclusion in survey, exclusion criteria).	Section 2.1
Sample characteristics	6b	Describe the sampling techniques used (e.g., single stage or multistage sampling, simple random sampling, stratified sampling, cluster sampling, convenience sampling). Specify the locations of sample participants whenever clustered sampling was applied.	Section 2.1
Sample characteristics	6c	Provide information on sample size, along with details of sample size calculation.	Section 2.1; Section 4.2 — no a priori sample-size calculation was performed; the achieved n is reported and its power discussed
Sample characteristics	6d	Describe how representative the sample is of the study population (or target population if possible), particularly for population-based surveys.	Section 2.1; Section 4.2
Survey administration	7a	Provide information on modes of questionnaire administration, including the type and number of contacts, the location where the survey was conducted (e.g., outpatient room or by use of online tools, such as SurveyMonkey).	Section 2.1
Survey administration	7b	Provide information of survey's time frame, such as periods of recruitment, exposure, and follow-up days.	Section 2.1
Survey administration	7c	Provide information on the entry process: For non-web-based surveys, provide approaches to minimize human error in data entry. For web-based surveys, provide approaches to prevent "multiple participation" of participants.	Section 2.2 — no technical control against multiple submissions; closed-item response patterns were screened for duplicates and none was found
Study preparation	8	Describe any preparation process before conducting the survey (e.g., interviewers' training process, advertising the survey).	Section 2.2 — reviewed internally, not externally piloted
Ethical considerations	9a	Provide information on ethical approval for the survey if obtained, including informed consent, institutional review board [IRB] approval, Helsinki declaration, and good clinical practice [GCP] declaration (as appropriate).	Section 2.4; Institutional Review Board Statement

Ethical considerations	9c / 9b	Provide information about survey anonymity and confidentiality and describe what mechanisms were used to protect unauthorized access.	Section 2.4; Institutional Review Board Statement; Data Availability Statement
Statistical analysis	10a	Describe statistical methods and analytical approach. Report the statistical software that was used for data analysis.	Section 2.3
Statistical analysis	10b	Report any modification of variables used in the analysis, along with reference (if available).	Section 2.3
Statistical analysis	10c	Report details about how missing data was handled. Include rate of missing items, missing data mechanism (i.e., missing completely at random [MCAR], missing at random [MAR], or missing not at random [MNAR]), and methods used to deal with missing data (e.g., multiple imputation).	Section 2.2 — all closed items in blocks C, D and E4 were required, so there are no missing data on the rated items; the ranking grids accepted up to five ranks and all supplied ranks were scored
Statistical analysis	10d	State how non-response error was addressed.	Not applicable — there was no sampling frame, so non-response could not be quantified; the limitation is stated in Section 2.1 and Section 4.2
Statistical analysis	10e	For longitudinal surveys, state how loss to follow-up was addressed.	Not applicable — cross-sectional design
Statistical analysis	10f	Indicate whether any methods such as weighting of items or propensity scores have been used to adjust for non-representativeness of the sample.	Not applicable — no weighting or propensity adjustment was applied
Statistical analysis	10g	Describe any sensitivity analysis conducted.	Section 2.3; Section 3.3; Table S2
Respondent characteristics	11a	Report numbers of individuals at each stage of the study. Consider using a flow diagram, if possible.	Section 2.1
Respondent characteristics	11b	Provide reasons for non-participation at each stage, if possible.	Not applicable
Respondent characteristics	11c	Report response rate, present the definition of response rate or the formula used to calculate response rate.	Not applicable — the questionnaire was distributed through professional networks with no fixed denominator, so a response rate cannot be calculated; stated in Section 2.1
Respondent characteristics	11d	Provide information to define how unique visitors are determined. Report number of unique visitors along with relevant proportions (e.g., view proportion, participation proportion, completion proportion).	Not applicable
Descriptive results	12	Provide characteristics of study participants, as well as information on potential confounders and assessed outcomes.	Section 2.1; Section 3.1
Main findings	13a	Give unadjusted estimates and, if applicable, confounder-adjusted estimates along with 95% confidence intervals and p values.	Sections 3.2–3.9; Table 1; Tables S1–S5; Figure S1
Main findings	13b	For multivariable analysis, provide information on the model building process, model fit statistics, and model assumptions (as appropriate).	Section 2.3; Section 3.4 (estimation method, slope with SE and CI, variance components)
Main findings	13c	Provide details about any sensitivity analysis performed. If there are considerable amount of missing data, report sensitivity analyses comparing the results of complete cases with that of the imputed dataset (if possible).	Section 3.3; Section 3.9
Limitations	14	Discuss the limitations of the study, considering sources of potential biases and imprecisions, such as non-representativeness of sample, study design, important uncontrolled confounders.	Section 4.2
Interpretations	15	Give a cautious overall interpretation of results, based on potential biases and imprecisions and suggest areas for future research.	Section 4; Section 5
Generalizability	16	Discuss the external validity of the results.	Section 4.2; Section 5
Role of the funding source	17	State whether any funding organization has had any roles in the survey's design, implementation, and analysis.	Funding
Conflict of interest	18	Declare any potential conflict of interest.	Conflicts of Interest
Acknowledgements	19	Provide names of organizations/persons that are acknowledged along with their contribution to the research.	Acknowledgments
