# Supplementary material for: Trust, Access, and the Division of Responsibility for Vaccine Confidence in Central and Eastern Europe: Paired Own-Country and Regional Ratings from a Multi-Stakeholder Professional Panel
Source: Vaccines (Basel). 2026 Sep 17;14(9):819. doi: 10.3390/vaccines14090819 (PMC13611672; doi:10.3390/vaccines14090819)
Supplement: Supplementary file 1 [file vaccines-14-00819-s001.zip › suplement/Supplementary_Tables_S1-S5_Figure_S1.pdf]

## Supplementary Tables S1–S5 and Figure S1

Table S1, Table S5 and Figure S1 report the material moved out of the article at the reviewers' request; Table S2 gives the fifteen item-level own-country versus region comparisons with unadjusted and Benjamini–Hochberg adjusted p-values; Tables S3 and S4 order the sixteen candidate interventions separately by expected impact and by feasibility within 12 to 24 months, to complement Table 1 (ordered by the impact–feasibility gap) and Figure 4. All means are across the 62 respondents on 1–5 scales.

### Table S1. Barrier ratings by item: mean at own-country and region level, and proportion rating the item serious or critical (4/5) at country level (N = 62).

*Items are ordered by own-country mean. Item codes are those used in Figures 1 and 2.*

| Barrier | Country mean | Region mean | % 4-5 (country) |
|---|---|---|---|
| C2.1 Digital misinformation and disinformation | 4.24 | 3.95 | 81% |
| C1.2 Distrust in government or political institutions | 3.84 | 3.47 | 65% |
| C4.1 Concerns about vaccine safety, especially newer technologies | 3.82 | 3.61 | 58% |
| C2.2 Low health and vaccine literacy | 3.69 | 3.44 | 61% |
| C4.4 Lack of culturally adapted communication for vulnerable groups | 3.48 | 3.42 | 47% |
| C1.4 Inconsistent or politically charged public communication | 3.40 | 3.21 | 48% |
| C1.1 Distrust in public health authorities and institutions | 3.40 | 3.34 | 48% |
| C2.3 Politicization of vaccine topics | 3.31 | 3.27 | 45% |
| C1.3 Insufficient or weak HCP vaccine recommendation | 3.19 | 3.02 | 35% |
| C4.2 Previous negative experiences with the health system | 3.11 | 3.15 | 39% |
| C3.3 Healthcare workforce shortages / limited counselling time | 2.95 | 3.02 | 34% |
| C3.4 Underdeveloped adult / life-course vaccination policies | 2.87 | 3.08 | 29% |
| C4.3 Social and family-level pressure | 2.85 | 3.10 | 26% |
| C3.1 Geographic or logistical access barriers | 2.76 | 3.02 | 24% |
| C3.2 Financial barriers (out-of-pocket, lack of reimbursement) | 2.61 | 3.03 | 27% |

### Table S2. Within-respondent comparison of own-country and regional ratings for each of the fifteen barriers: unadjusted and Benjamini–Hochberg adjusted p-values (N = 62).

*Items are listed in the order of Table S1. Columns give the mean own-country and regional ratings, the mean of the own-country minus regional difference computed per respondent, the two-sided Wilcoxon signed-rank test (ties at zero excluded), the rank-biserial correlation as effect size, and the Benjamini–Hochberg adjusted p-value (q). These are the fifteen exploratory comparisons described in Section 3.3; the correction was applied across these fifteen comparisons only, as one family, and no comparison reaches q < 0.05 (smallest q = 0.051, for C1.2 and C3.2). The primary test of the asymmetry is the difference-of-differences interaction reported in Section 3.3, a single pre-specified comparison that is not part of this family.*

| Barrier | Own-country mean | Region mean | Mean difference (own minus region) | Wilcoxon p | Rank-biserial r | BH-adjusted q |
|---|---|---|---|---|---|---|
| C2.1 Digital misinformation and disinformation | 4.24 | 3.95 | +0.29 | 0.035 | +0.44 | 0.131 |
| C1.2 Distrust in government or political institutions | 3.84 | 3.47 | +0.37 | 0.007 | +0.56 | 0.051 |
| C4.1 Concerns about vaccine safety, especially newer technologies | 3.82 | 3.61 | +0.21 | 0.075 | +0.37 | 0.160 |
| C2.2 Low health and vaccine literacy | 3.69 | 3.44 | +0.26 | 0.054 | +0.36 | 0.145 |
| C4.4 Lack of culturally adapted communication for vulnerable groups | 3.48 | 3.42 | +0.06 | 0.698 | +0.08 | 0.748 |
| C1.4 Inconsistent or politically charged public communication | 3.40 | 3.21 | +0.19 | 0.207 | +0.23 | 0.310 |
| C1.1 Distrust in public health authorities and institutions | 3.40 | 3.34 | +0.06 | 0.627 | +0.11 | 0.748 |
| C2.3 Politicization of vaccine topics | 3.31 | 3.27 | +0.03 | 0.865 | +0.03 | 0.865 |
| C1.3 Insufficient or weak HCP vaccine recommendation | 3.19 | 3.02 | +0.18 | 0.111 | +0.30 | 0.208 |

| | | | | | | |
|---|---|---|---|---|---|---|
| C4.2 Previous negative experiences with the health system | 3.11 | 3.15 | -0.03 | 0.691 | -0.08 | 0.748 |
| C3.3 Healthcare workforce shortages / limited counselling time | 2.95 | 3.02 | -0.06 | 0.597 | -0.10 | 0.748 |
| C3.4 Underdeveloped adult / life-course vaccination policies | 2.87 | 3.08 | -0.21 | 0.143 | -0.27 | 0.238 |
| C4.3 Social and family-level pressure | 2.85 | 3.10 | -0.24 | 0.032 | -0.40 | 0.131 |
| C3.1 Geographic or logistical access barriers | 2.76 | 3.02 | -0.26 | 0.058 | -0.35 | 0.145 |
| C3.2 Financial barriers (out-of-pocket, lack of reimbursement) | 2.61 | 3.03 | -0.42 | 0.004 | -0.56 | 0.051 |

## Table S3. Interventions ordered by expected impact.

*Highest expected impact first; exact ties (D2.1 and D4.2; D3.3 and D4.4) are listed in item-code order. Gaps are differences of the unrounded means, as in Table 1. The quadrant classification uses the medians of the two axes (expected impact 3.98, feasibility 3.45), as in Figure 4.*

| Intervention | Expected impact | Feasibility | Gap | Quadrant |
|---|---|---|---|---|
| D1.1 Continuing-education for primary care on vaccine communication | 4.21 | 3.74 | +0.47 | High impact / high feasibility |
| D2.1 Local campaigns led by trusted community figures | 4.13 | 3.65 | +0.48 | High impact / high feasibility |
| D4.2 Adoption of life-course immunization policies | 4.13 | 3.29 | +0.84 | High impact / lower feasibility |
| D3.3 Real-time monitoring of vaccine narratives on social media | 4.10 | 3.39 | +0.71 | High impact / lower feasibility |
| D4.4 Increased institutional transparency on vaccine decisions | 4.10 | 3.11 | +0.98 | High impact / lower feasibility |
| D4.1 Integration of vaccination into primary care and schools | 4.05 | 3.42 | +0.63 | High impact / lower feasibility |
| D3.2 Proactive prebunking and counter-narrative campaigns | 4.02 | 3.48 | +0.53 | High impact / high feasibility |
| D2.3 Outreach and culturally adapted programs for vulnerable populations | 4.00 | 3.40 | +0.60 | High impact / lower feasibility |
| D2.4 Tailored materials co-created with vulnerable communities | 3.95 | 3.50 | +0.45 | Lower impact / high feasibility |
| D1.2 Communication toolkits and decision aids for clinicians | 3.90 | 3.81 | +0.10 | Lower impact / high feasibility |
| D3.1 Audience-segmented messaging | 3.84 | 3.58 | +0.26 | Lower impact / high feasibility |
| D4.3 Investment in interoperable digital data systems | 3.73 | 2.98 | +0.74 | Lower impact / lower feasibility |
| D1.3 Expanded role for pharmacists in adult vaccination | 3.61 | 3.27 | +0.34 | Lower impact / lower feasibility |
| D1.4 Peer support / mentoring networks for healthcare workers | 3.61 | 3.32 | +0.29 | Lower impact / lower feasibility |
| D2.2 Networks of vaccination ambassadors | 3.60 | 3.52 | +0.08 | Lower impact / high feasibility |
| D3.4 SMS/app reminders and digital scheduling tools | 3.58 | 3.50 | +0.08 | Lower impact / high feasibility |

## Table S4. Interventions ordered by feasibility within 12–24 months.

*Most feasible first; exact ties (D2.4 and D3.4) are listed in item-code order; columns and quadrants as in Table S3.*

| Intervention | Expected impact | Feasibility | Gap | Quadrant |
|---|---|---|---|---|
| D1.2 Communication toolkits and decision aids for clinicians | 3.90 | 3.81 | +0.10 | Lower impact / high feasibility |
| D1.1 Continuing-education for primary care on vaccine communication | 4.21 | 3.74 | +0.47 | High impact / high feasibility |

| | | | | |
|---|---|---|---|---|
| D2.1 Local campaigns led by trusted community figures | 4.13 | 3.65 | +0.48 | High impact / high feasibility |
| D3.1 Audience-segmented messaging | 3.84 | 3.58 | +0.26 | Lower impact / high feasibility |
| D2.2 Networks of vaccination ambassadors | 3.60 | 3.52 | +0.08 | Lower impact / high feasibility |
| D2.4 Tailored materials co-created with vulnerable communities | 3.95 | 3.50 | +0.45 | Lower impact / high feasibility |
| D3.4 SMS/app reminders and digital scheduling tools | 3.58 | 3.50 | +0.08 | Lower impact / high feasibility |
| D3.2 Proactive prebunking and counter-narrative campaigns | 4.02 | 3.48 | +0.53 | High impact / high feasibility |
| D4.1 Integration of vaccination into primary care and schools | 4.05 | 3.42 | +0.63 | High impact / lower feasibility |
| D2.3 Outreach and culturally adapted programs for vulnerable populations | 4.00 | 3.40 | +0.60 | High impact / lower feasibility |
| D3.3 Real-time monitoring of vaccine narratives on social media | 4.10 | 3.39 | +0.71 | High impact / lower feasibility |
| D1.4 Peer support / mentoring networks for healthcare workers | 3.61 | 3.32 | +0.29 | Lower impact / lower feasibility |
| D4.2 Adoption of life-course immunization policies | 4.13 | 3.29 | +0.84 | High impact / lower feasibility |
| D1.3 Expanded role for pharmacists in adult vaccination | 3.61 | 3.27 | +0.34 | Lower impact / lower feasibility |
| D4.4 Increased institutional transparency on vaccine decisions | 4.10 | 3.11 | +0.98 | High impact / lower feasibility |
| D4.3 Investment in interoperable digital data systems | 3.73 | 2.98 | +0.74 | Lower impact / lower feasibility |

**Table S5. Mean domain ratings by stakeholder group (barrier severity and intervention impact, 1–5), for the two largest groups and the full sample.**

*Civil-society and patient-organisation representatives (n = 25) and clinical health professionals (n = 21); see Section 3.9 for the comparison.*

| Domain | Civil society (n=25) | Clinical HCP (n=21) | All (N=62) |
|---|---|---|---|
| C1 Trust in institutions | 3.32 | 3.54 | 3.46 |
| C2 Information & literacy | 3.75 | 3.60 | 3.75 |
| C3 Access & system | 2.92 | 2.55 | 2.80 |
| C4 Attitudes, safety, culture | 3.36 | 3.27 | 3.32 |
| D1 Clinician education & tools | 3.98 | 3.76 | 3.83 |
| D2 Community & outreach | 3.97 | 3.80 | 3.92 |
| D3 Messaging & monitoring | 3.90 | 3.68 | 3.88 |
| D4 Structural & policy | 4.11 | 4.02 | 4.00 |

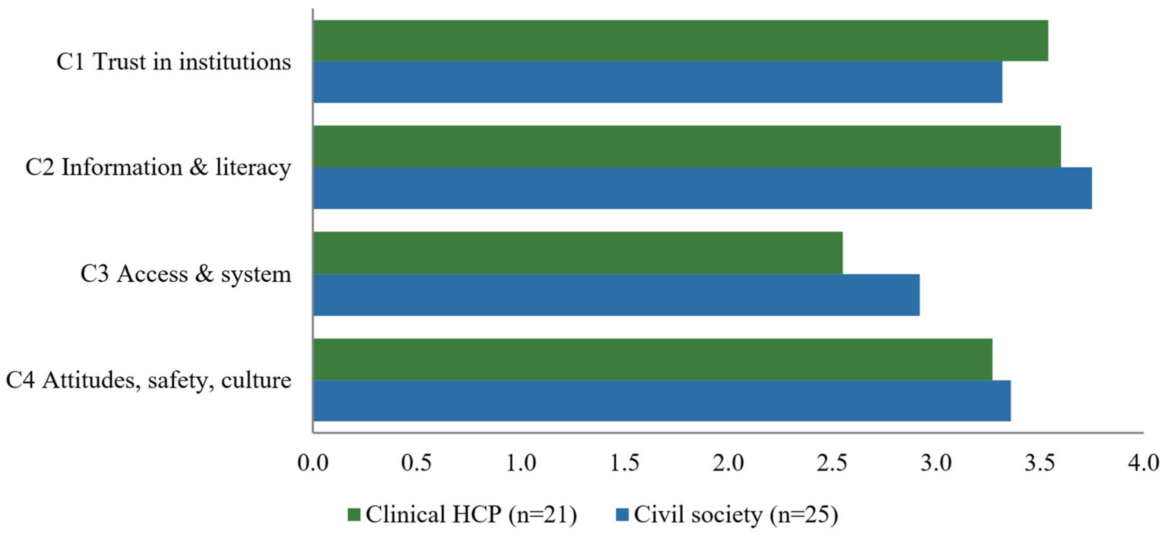


**Figure S1. Barrier severity by thematic domain, civil-society and patient-organisation representatives (n = 25) versus clinical health professionals (n = 21); mean own-country ratings.**
